# Identification of cuproptosis-related subtypes in lung adenocarcinoma and its potential significance

**DOI:** 10.3389/fphar.2022.934722

**Published:** 2022-10-03

**Authors:** Shize Pan, Congkuan Song, Heng Meng, Ning Li, Donghang Li, Bo Hao, Zilong Lu, Qing Geng

**Affiliations:** Department of Thoracic Surgery, Renmin Hospital of Wuhan University, Wuhan, China

**Keywords:** cuproptosis, lung adenocarcinoma, tumor microenvironment, immunotherapy, drug sensitivity

## Abstract

Cuproptosis is a novel and unique cell death mode that has attracted significant interest in recent years. Little is currently known about whether cuproptosis-related genes (CRGs) are associated with the pathophysiology and survival of patients with lung adenocarcinoma (LUAD). The present study sought to characterize the transcriptional and genetic alteration of CRGs in LUAD and its potential significance in the tumor microenvironment and predicting the prognosis of LUAD. The secondary eventual aim was to study the role of CRGs in predicting immunotherapy response and its clinical value combined with the TNM stage. We found that several CRGs, including FDX1, DLD, SLC31A1, and MTF1, were enriched in macrophages in our single-cell RNA-seq data. Three distinct molecular subtypes were identified and correlated with clinicopathological characteristics, prognosis, biological pathways, and tumor microenvironment (TME) in LUAD. We developed a cuproptosis-related gene score (CRG_score) and validated it in three independent cohorts and clinical subtypes. The low CRG_score group, characterized by a greater immune score, immunophenoscore (IPS), lower tumor immune dysfunction and exclusion (TIDE) score, and T-cell dysfunction score, had a better prognosis, suggesting that the low CRG_score group responded more favorably to immunotherapy, which was validated in the anti-PD-1/L1 immunotherapy cohort (IMvigor210). In contrast, the high CRG_score group was more sensitive to targeted therapy and chemotherapy, with a higher cancer stem cell (CSC) index and lower half-maximal inhibitory concentration (IC50) for many drugs. Given the established crosstalk between CRG_score and tumor TNM stage, we developed an accurate nomogram for clinical application of the CRG_score. Taken together, our rigorous and comprehensive examination of CRGs in LUAD identified their potential functions in TME, clinicopathological characteristics, drug sensitivity, and prognosis. These findings improve the current understanding of cuproptosis in LUAD, paving the way for more accurate prognosis assessment and tailored treatment for this patient population.

## Instruction

Lung cancer (LC) is one of the most prevalent types of cancer and the main cause of cancer-related mortality globally (Cancer Genome Atlas Research, 2014). LC is classified into two primary subtypes based on histologic type: small cell lung cancer (SCLC) and non-small cell lung cancer (NSCLC), which account for 15% and 85% of LCs, respectively (Chen et al., 2016). Current evidence suggests that the prevalence of LUAD has increased in recent years compared to other lung cancer subtypes (Meza et al., 2015). TNM staging of tumors has long been widely used to predict the prognosis of LC, but it has been shown that there are differences in survival in patients with the same stage of lung cancer due to tumor heterogeneity (Hensing et al., 2014). As a result, a biomarker capable of reliably predicting the prognosis of lung cancer is urgently needed. Notwithstanding that substantial breakthroughs have been achieved in immunotherapy for advanced lung cancer, there is still a lack of reliable clinical biomarkers to identify which lung cancer patient populations are most likely to derive benefit (Reck et al., 2019; Gadgeel et al., 2020). Accordingly, it is essential to find a biomarker that can accurately predict the response to immunotherapy of lung cancer patients.

Copper levels have been demonstrated to act as a “double-edged sword” for cell viability. On the one hand, low intracellular copper concentrations are essential for cellular homeostasis. On the other hand, the accumulation of free intracellular copper is detrimental to cells, and occasionally even moderate intracellular copper concentrations can be toxic and eventually result in cell death (Kim et al., 2008). Copper-induced cell death, also known as cuproptosis, is a unique mechanism of cell death distinct from documented programmed cell deaths (PCD), such as apoptosis, pyroptosis, necroptosis, and ferroptosis. Copper can reportedly combine with thioredoxin in the tricarboxylic acid cycle (TCA) of mitochondrial respiration, resulting in abnormal thioredoxin oligomerization, reducing the levels of iron-sulfur (Fe-S) clusters, and causing proteotoxic stress response and ultimately cuproptosis (Tsvetkov et al., 2022). Recent research indicated that imbalanced copper homeostasis could impair tumor growth and result in irreparable harm (Li, 2020). A previous study also demonstrated that copper could induce activation of various cell death pathways, including apoptosis and autophagy, as well as the formation of reactive oxygen species, proteasome inhibition, and anti-angiogenesis (Jiang et al., 2022). Therefore, cuproptosis may play an important role in tumor development. FDX1, DLAT, and LIAS are reportedly essential for cuproptosis (Tsvetkov et al., 2022). In a previous study, knockdown of FDX1 was found to alter tumor cell metabolism, thereby affecting tumor-associated inflammation and changes in the immune microenvironment (Zhang et al., 2021a). Moreover, DLAT could acetylate the k76 site of 6-phosphogluconate dehydrogenase (6PGD), thereby promoting the proliferation and growth of H1299 lung cancer cells (Shan et al., 2014). Besides, LIAS has been shown to regulate HIF-1α activity and may have broad implications for epigenetic regulation and tumorigenesis (Burr et al., 2016). However, the synergistic effects of multiple CRGs in lung adenocarcinoma have not been reported.

In this work, we aimed to evaluate the molecular alterations and clinical relevance of CRGs in LUAD. Our data emphasized the significance of CRGs in the pathogenesis of LUAD and established the groundwork for the accurate prediction of LUAD prognosis and immunotherapy response.

## Materials and methods

### Data sources

**FIGURE 1:**
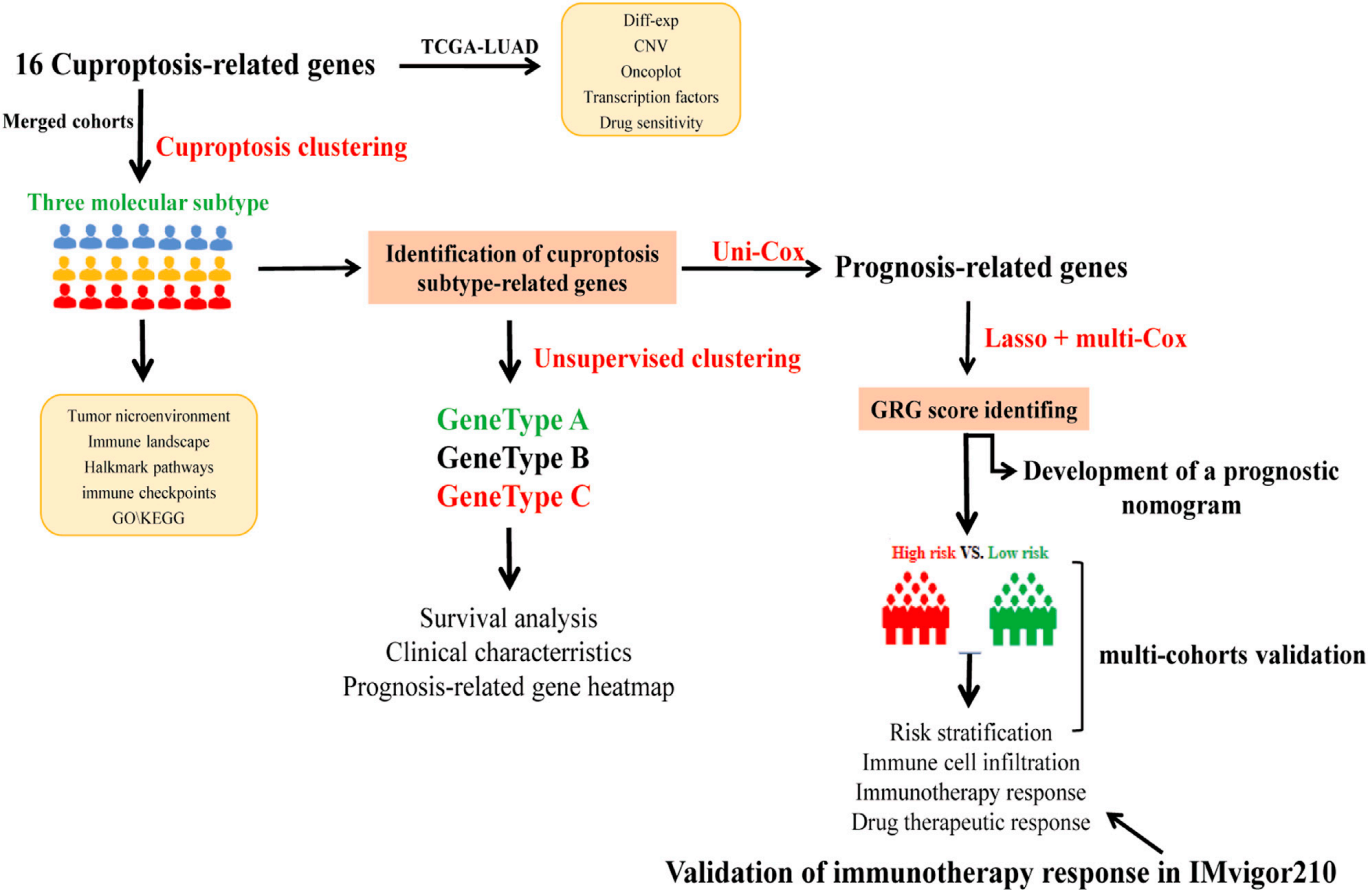
The flow diagram showed the entire analytical process of the study.

The study’s flow diagram is depicted in Figure 1. We retrieved 16 CRGs from a published article (Supplementary Table S1) (Tsvetkov et al., 2022). The Gene Expression Omnibus (GEO) (https://www.ncbi.nlm.nih.gov/geo/) and The Cancer Genome Atlas (TCGA) databases were utilized to obtain RNAseq data and corresponding clinical characteristics of LUAD. Finally, a total of 1972 LUAD samples were enrolled, including 30 from GSE29013 (Xie et al., 2011), 85 from GSE30219 (Rousseaux et al., 2013), 226 from GSE31210 (Okayama et al., 2012), 106 from GSE37745 (Botling et al., 2013), 127 from GSE50081 (Der et al., 2014), 443 from GSE68465 (Director’s Challenge Consortium for the Molecular Classification of Lung Adenocarcinoma et al., 2008), 442 from GSE72094 (Schabath et al., 2016) and 513 from TCGA-LUAD cohort. As previously described, RNAseq data (transcripts per kilobase million, TPM) could be combined with RNAseq in the microarray by batch correction (Conesa et al., 2016; Song et al., 2021). The “ComBat” method was used to avoid the batch effect. The batch effect was eliminated after the combination, as shown in Supplementary Figure S1. 1972 LUAD samples from the merged cohort were retained for subsequent analysis. The anti-PD1/PD-L1 treatment cohort (Imvigor210) was also collected from a previously published study (Mariathasan et al., 2018). Moreover, we downloaded the TCGA somatic mutation from GDC [GDC(cancer.gov)] and the copy number variation (CNV) from UCSC Xena (https://xenabrowser.net).

### Data sources for each scoring system

TIDE, T cell dysfunction, and T cell exclusion were obtained from the TIDE website (http://tide.dfci.harvard.edu). The IPS was derived from a published article (Charoentong et al., 2017). RNAs served as a measure for the degree of similarity between tumor cells and stem cells and thus could be used to quantify cancer stem cells (CSCs) (Malta et al., 2018). RNAs were obtained from the UCSC website [UCSC Xena(xenabrowser.net)].

### Establishment of the regulatory network between transcription factors and cuproptosis-related genes

Transcription factor (TF) binding motifs for humans were acquired from the RcisTarget database (https://resources.aertslab.org/cistarget/). The network was constructed using the “visNetwork” R package (Peng et al., 2019).

### Single-cell RNA-Seq data processing

Our single-cell RNA-Seq data were obtained from three LUAD patients and three control patients. All patients signed an informed consent form and met the inclusion criteria. The single-cell RNA-seq data were deposited at NODE under the project ID: OEP000943 (https://www.biosino.org/node/). The “Seurat” R package was utilized for downstream analysis of single-cell RNA profiles (Hao et al., 2021). The R package “singleR” was used to automatically annotate cell subsets.

### Consensus clustering analysis of cuproptosis-related genes

LUAD samples were categorized using unsupervised clustering analysis. This clustering was performed by applying the following criteria. The curve of cumulative distribution function (CDF) was plotted by gradually increasing the k value and there were no groups with a small sample size. Finally, the optimal number of clusters was characterized by an increased intra-group correlation and decreased inter-group correlation. The above procedure was conducted with the R package “ConsensusClusterPlus” and repeated 1,000 times to ensure the classification’s stability (Wilkerson and Hayes, 2010).

### Establishment and validation of the cuproptosis-related gene_score

DEGs among different cuproptosis molecular subtypes were identified using the screening criteria: fold-change of 1.2 and an adjusted *p*-value of 0.05. Using univariate/multivariate Cox regression analysis, the prognostic genes most strongly related to LUAD OS were determined. To minimize the risk of overfitting, the prognosis-related DEGs were included in the LASSO regression analysis, and the scoring signature was constructed using multivariate cox regression analysis as follows: CRG_score = (Expi * coefi), where Coefi and Expi denote the risk coefficient and expression of each gene, respectively. The merged cohort and all independent validation sets were categorized as high- or low-risk groups based on the median risk score. Next, we validated the value of the CRGs signature in mortality risk identification, classification, and prognostic ability in the three independent datasets.

### Tissue samples

Six pairs of LUAD and adjacent non-tumor tissues were obtained from patients with LUAD at the Renmin Hospital of Wuhan University. All participants in this study provided written informed consent. The Ethics Committee of Wuhan University’s Renmin Hospital approved this study.

### Real-time fluorescence quantitative PCR

Total RNA was extracted from LUAD patient tissues using TRIpure Total RNA Extraction Reagent (ELK Biotechnology, EP013), and cDNA was synthesized using EntiLink™ first Strand cDNA Synthesis Kit (ELK Biotechnology, EP003). SYBR-Green assays (ELK Biotechnology, EP001) were used to perform RT-qPCR. The expression levels of target genes were uniformly normalized to GAPDH. The primer sequences used for RT-qPCR in this study are listed in Supplementary Table S2.

### Estimation of drug sensitivity

Downloads from CellMiner (https://discover.nci.nih.gov/cellminer/home.do) included two data labeled “RNA: RNA-seq” and “Compound activity: DTP NCI-60". We further investigated the connection between FDA-approved drug Z scores with cuproptosis-related DEGs. A lower IC50 indicated higher drug sensitivity in cells. We computed the IC50 of commonly cancer-fighting drugs using the R package “pRRophetic” (Geeleher et al., 2014).

### Pathway and function enrichment analysis

Hallmark gene sets were obtained from the MSigDB database [GSEA(gsea-msigdb.org)], which included 50 marker gene lists that define biological states and processes (Supplementary Table S3). Gene set variation analysis (GSVA) (Hanzelmann et al., 2013), a nonparametric and unsupervised approach for examining the biological pathways of various populations, was performed to analyze these 50 biological pathways. Metascape (https://metascape.org/gp/index.html#/main/step1) is a web-based portal created to perform pathway and function enrichment analyses on gene lists (Zhou et al., 2019). The DEGs among molecular subgroups were analyzed using Metascape, Gene Ontology (GO), and the Kyoto Encyclopedia of Genes and Genomes (KEGG).

### Estimation of the tumor microenvironment and immune landscape

TME refers to the local environment composed of tumor cells, stromal cells, immune cells, cytokines, and chemokines (Balkwill et al., 2012). To obtain a better understanding of TME in various subgroups, we assessed immune cells using various methods, including single sample GSEA (ssGSEA) (Barbie et al., 2009), TIMER (Li et al., 2017), CIBRESORT (Newman et al., 2015), QUANTISEQ (Finotello et al., 2019), MCPcounter (Becht et al., 2016), XCELL (Aran et al., 2017), and EPIC (Racle and Gfeller, 2020). The ESTIMATE algorithm (Becht et al., 2016) was used to estimate the amount of stromal and immune cells in malignant tumors and compute immune scores, stromal scores, and estimate scores.

### Establishment and validation of a prognostic nomogram

A predictive nomogram was constructed based on clinical variables and risk scores (Iasonos et al., 2008). The nomogram scoring system was used to assign a score to each variable, and the overall score was calculated by summing the scores of all variables. The predictive value of the nomogram was compared with the TNM stage for the 1, 3, 5, and 10-year survival probability using time-dependent ROC. The nomogram calibration plot was used to compare the predicted 1-, 3-, and 5-year survival events to the actual outcomes.

### Statistical analysis

A *p*-value <0.05 was statistically significant. R 4.0.2 (https://www.r-project.org) and *OriginPro2021* were utilized to analyze data and generate tables and figures.

## Results

### Transcriptional and genetic alterations associated with cuproptosis and transcription factor regulation and drug targets

**FIGURE 2:**
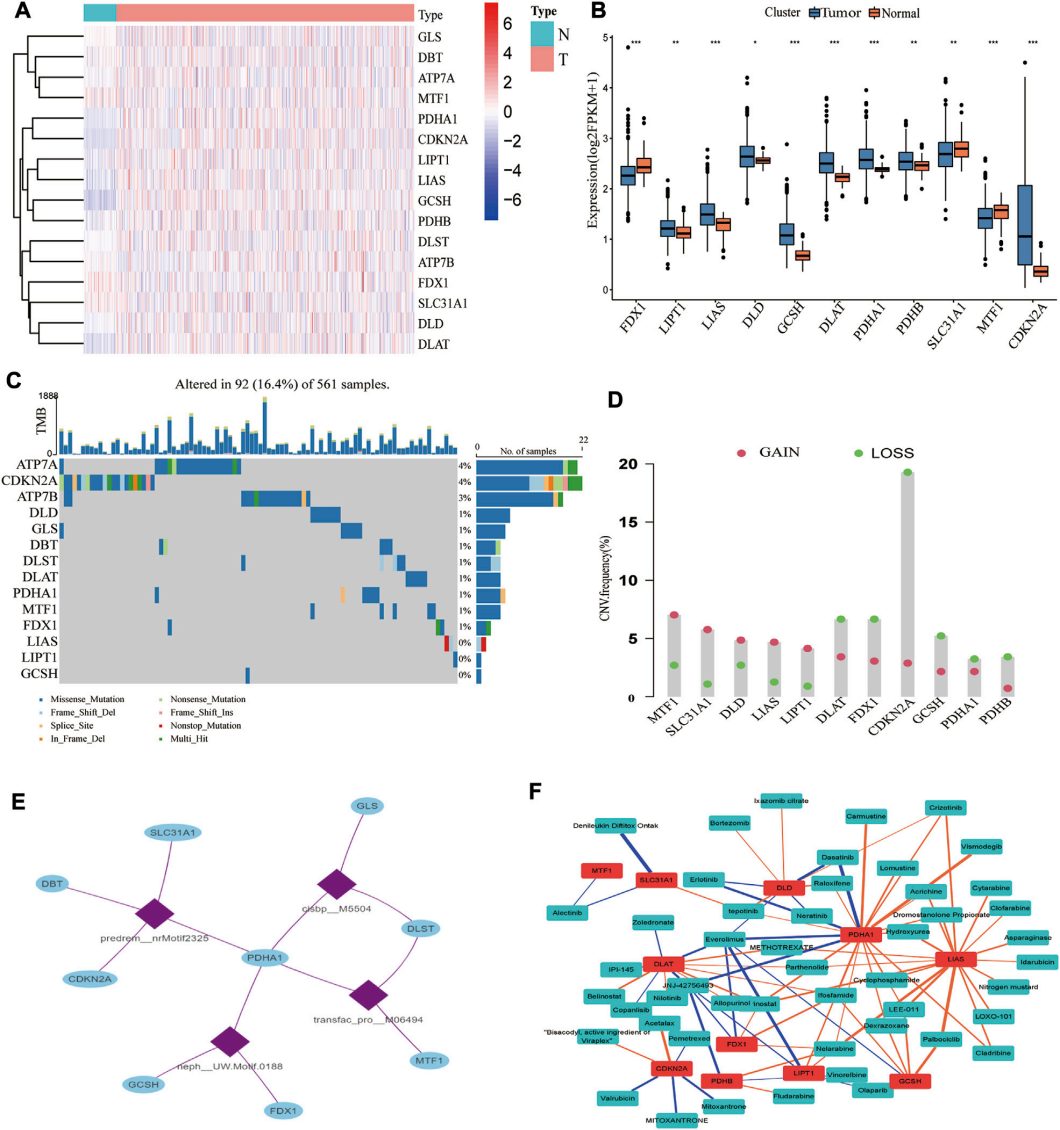
Transcriptional and genetic alterations of CRGs, regulatory networks of transcription factors, and sensitive drugs. **(A)** Heatmap showing the expression pattern of 16 CRGs in LUAD (N: tumor, T: tumor; red represents high expression, blue represents low expression). **(B)** 11 CRGs were differentially expressed in normal and tumor tissues. **(C)** Somatic mutations of CRGs. **(D)** The mutation frequency of CNV is prevalent in CRGs. **(E)** The top 4 transcription factor motifs that mostly possibly regulate the CRGs. **(F)** Correlation of 11 differentially expressed CRGs with sensitive drug Z scores (blue: negative correlation, orange: positive correlation; thickness of the line represents the strength of the correlation).

Cancer is widely acknowledged as a highly heterogeneous disease with distinct gene expression patterns. Our study showed considerable differences in genetic profiles and expression levels of CRGs, implying that CRGs may potentially participate in the pathogenesis of LUAD. We examined the differential expression of 16 CRGs in the TCGA dataset. As seen in Figure 2A, 11 CRGs were differentially expressed between tumor and normal tissues, with eight upregulated and three downregulated in LUAD (Figure 2B; Supplementary Table S4). FDX1, LIPT1, and DLAT were related to prognosis, with FDX1 and LIPT1 being independent prognostic factors (Supplementary Figures S2A,B). Further research revealed that decreased FDX1 and DLAT expression was associated with improved OS, while increased LIPT1 expression was associated with worse OS (Supplementary Figures S2C–E). Intriguingly, based on our single-cell RNA-seq data, we classified TME cells in lung cancer into seven main cell types and discovered that FDXL, DLD, SLC3A1, and PDHA1 were differentially expressed in macrophages (Supplementary Figure S3). A low mutation frequency of CRGs was observed (Figure 2C). Only 92 (16.4%) of the 561 LUAD samples harbored 16 CRGs somatic mutations, with ATP7A and CDKN2A being the most frequent mutations. Subsequently, we analyzed the CNVs of all CRGs. MTF1, SLC31A1, DLD, LIAS, and LIPT1 CNVs were consistently increased, whereas DLAT, FDX1, CDKN2A, GCSH, PDHA1, and PDHB CNVs generally decreased in LUAD (Figure 2D). CNV gain in DLD, LIAS, and LIPT1 boosted gene expression, and CNV loss in FDX1 resulted in a substantially reduced gene expression. In comparison, other CRGs with high CNV loss or gain exhibited opposing expression patterns, suggesting that CNV is not the only significant factor impacting mRNA expression (Sebestyen et al., 2016). A previous study demonstrated that other factors, such as DNA methylation and transcription factors, may regulate gene expression (Koch et al., 2018). As a result, we further investigated the transcription factor motifs profile of CRGs and identified the top 4 related transcription factor motifs using the RcisTarget database (Figure 2E). Moreover, we found that the drug sensitivity of several compounds was substantially related to these 11 differentially expressed CRGs (Figure 2F; Supplementary Table S5).

### Validation of the expression levels of cuproptosis-related genes

The expression levels of 16 CRGs were measured in six LUAD tissues and six adjacent normal tissues by RT-qPCR. As demonstrated in Supplementary Figure S4 and Supplementary Table S6, the expression levels of LIPT1, LIAS, GCSH, DLAT, PDHA1, PDHB, and CDKN2A were elevated, while those of FDX1, SLC31A1 and MTF1 were downregulated in LUAD tissues compared to normal tissues.

### Identification of cuproptosis molecular subtypes in lung adenocarcinoma

**FIGURE 3:**
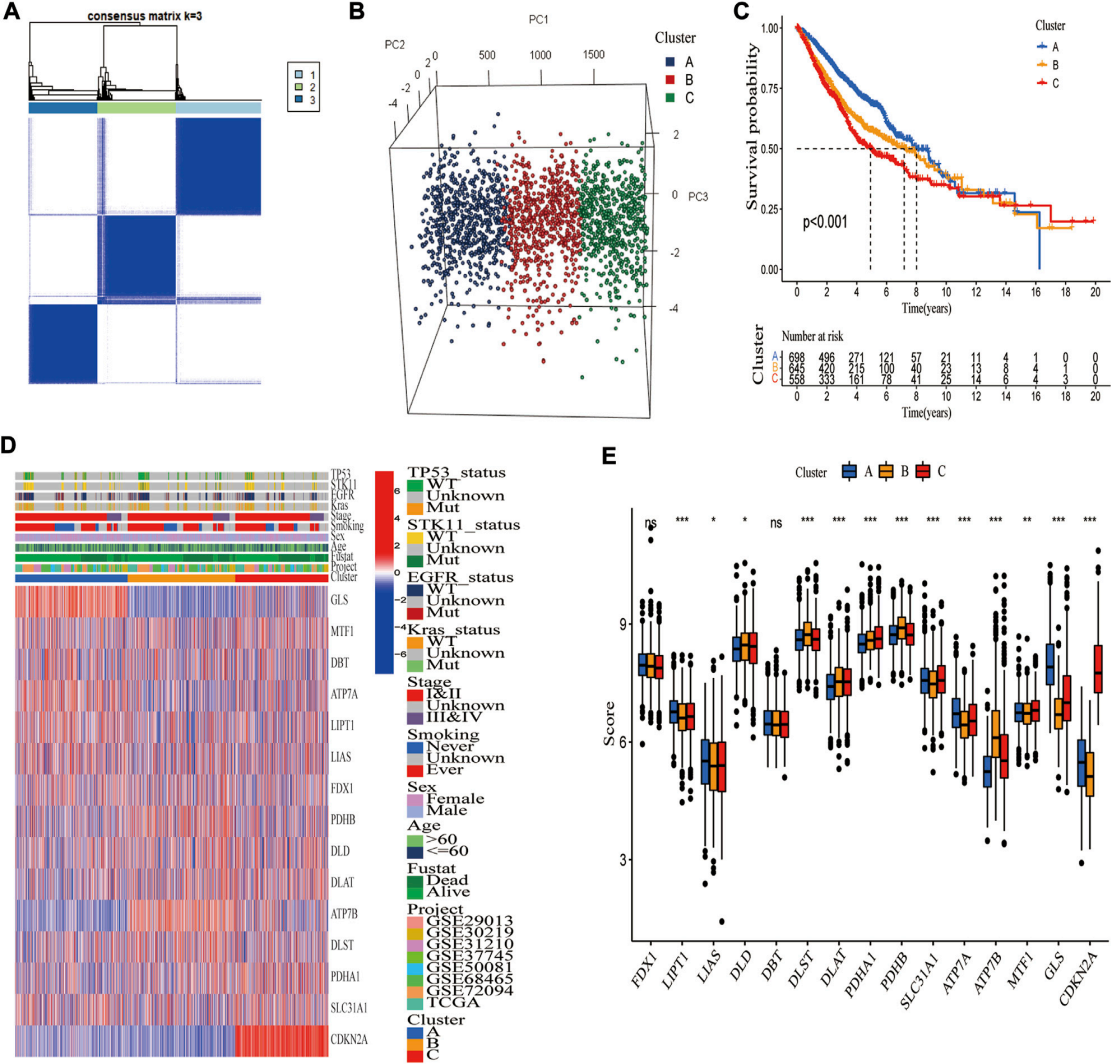
Identification of three cuproptosis molecular subtypes in the merged cohort. **(A)** Unsupervised consensus clustering identified three molecular subtypes of cuproptosis. **(B)** PCA verified that the merged cohort could be well classified into three molecular subtypes. **(C)** Kaplan-Meier analysis showed significant differences in overall survival between the three molecular subtypes. **(D)** Heatmap demonstrating differences in CRGs transcript and clinical features among three molecular subgroups (blue, low expression level; red, high expression level). **(E)** Thirteen CRGs were differentially expressed among three molecular subgroups. *, **, and ***, represent *p* < 0.05, *p* < 0.01, and *p* < 0.001, respectively.

For classification of cuproptosis into molecular subtypes based on CRGs, unsupervised cluster analysis was performed on these 1972 LUAD samples of the merged cohort. LUAD was classified into three cuproptosis molecular subtypes (k = 3) by increasing the clustering variable (k) from 2 to 9 (Supplementary Figure S5). These three subtypes were labeled as cluster A (*n* = 698), cluster B (*n* = 645), and cluster C (*n* = 558) (Figure 3A). Principal component analysis (PCA) verified the clustering results (Figure 3B), and tSNE yielded similar findings (Supplementary Figure S6). Kaplan-Meier analysis revealed that the three molecular subtypes had significantly different prognoses, with cluster A having the best survival outcomes (Figure 3C). The heatmap illustrated the clinical characteristics of various molecular subtypes, substantiating that LUAD can be categorized into three distinct molecular subtypes based on CRGs (Figure 3D). Furthermore, most CRGs were significantly differentially expressed among these three molecular subtypes (Figure 3E).

### Characteristics of tumor microenvironment cell infiltration and biological function in the cuproptosis molecular subtypes

**FIGURE 4:**
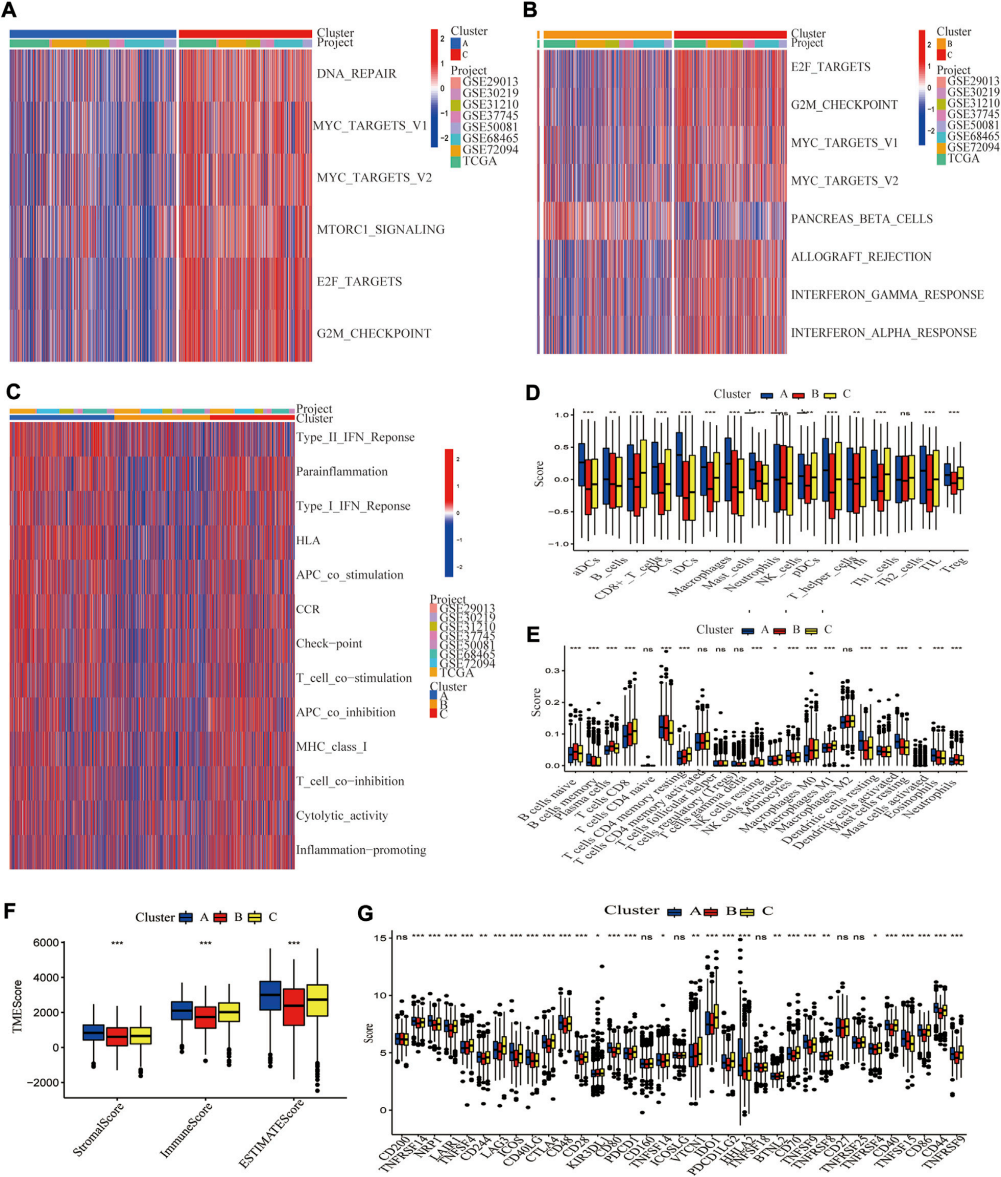
Differences in biological pathways and tumor microenvironment among three cuproptosis molecular subtypes of LUAD. GSVA analyzed the biological pathways of three cuproptosis subtypes. Red represents activation of biological pathways, and blue represents inhibition of biological pathways. **(A)** Cluster A vs. Cluster C; **(B)** Cluster B vs. Cluster C. **(C)** The immune-related pathway differences in Clusters A–C. Two algorithms demonstrated the abundance of TME cell infiltration for three cuproptosis subtypes **(D)** by ssGSEA; **(E)** by CEBERSORT. Statistical differences between the three clusters were analyzed by a one-way ANOVA test. **(F)** Tumor microenvironment scores between the three cuproptosis subtypes, including Stromal score, Immune score, and Estimated score. **(G)** Differential expression of different immune checkpoint genes among the three cuproptosis subtypes.*, **, and ***, represent *p* < 0.05, *p* < 0.01, and *p* < 0.001, respectively.

It was discovered that distinct molecular subtypes had different biological pathways and immunological landscapes, which may contribute to their drastically different survival probabilities. GSVA analysis was performed to investigate the functional and biological differences among these three molecular subtypes (Figures 4A–C). The results revealed that Cluster A was primarily enriched in immune activation-related pathways, such as T cell co-stimulation, APC co-stimulation, and Cytolytic activity, whereas Cluster B exhibited opposite results, with significant enrichment for lower immune activation pathways. Cluster C was enriched in more immunological pathways, such as T cell co-stimulation and inflammation-promoting pathways, several cell proliferation and oncogenic pathways, including MYC targets v1, MYC targets v2, G2M checkpoint, and E2F targets signaling pathways. We then employed ssGSEA of the “GSVA” R package to evaluate the enrichment scores of 16 immune cells (Supplementary Table S7). Activated DCs, Macrophages, Mast cells, Neutrophils, TIL, and Treg cells were the most prominent immune infiltrating cells in Cluster A. CD8^+^ T cells were the most enriched in Cluster C, while immune cells were the least enriched in Cluster B (Figure 4D). CIBERSORT yielded similar results to ssGSEA (Figure 4E). As predicted, Cluster A had the highest immune and stromal scores, while Cluster B had the lowest in TME (Figure 4F). What’s more, immune checkpoint genes were differentially expressed among these three molecular subtypes, with CTLA4 and PD1 (PDCD1) exhibiting higher expressions in Cluster C than in Clusters A and B (Figure 4G).

### Identification of cuproptosis phenotype-associated subtypes

**FIGURE 5:**
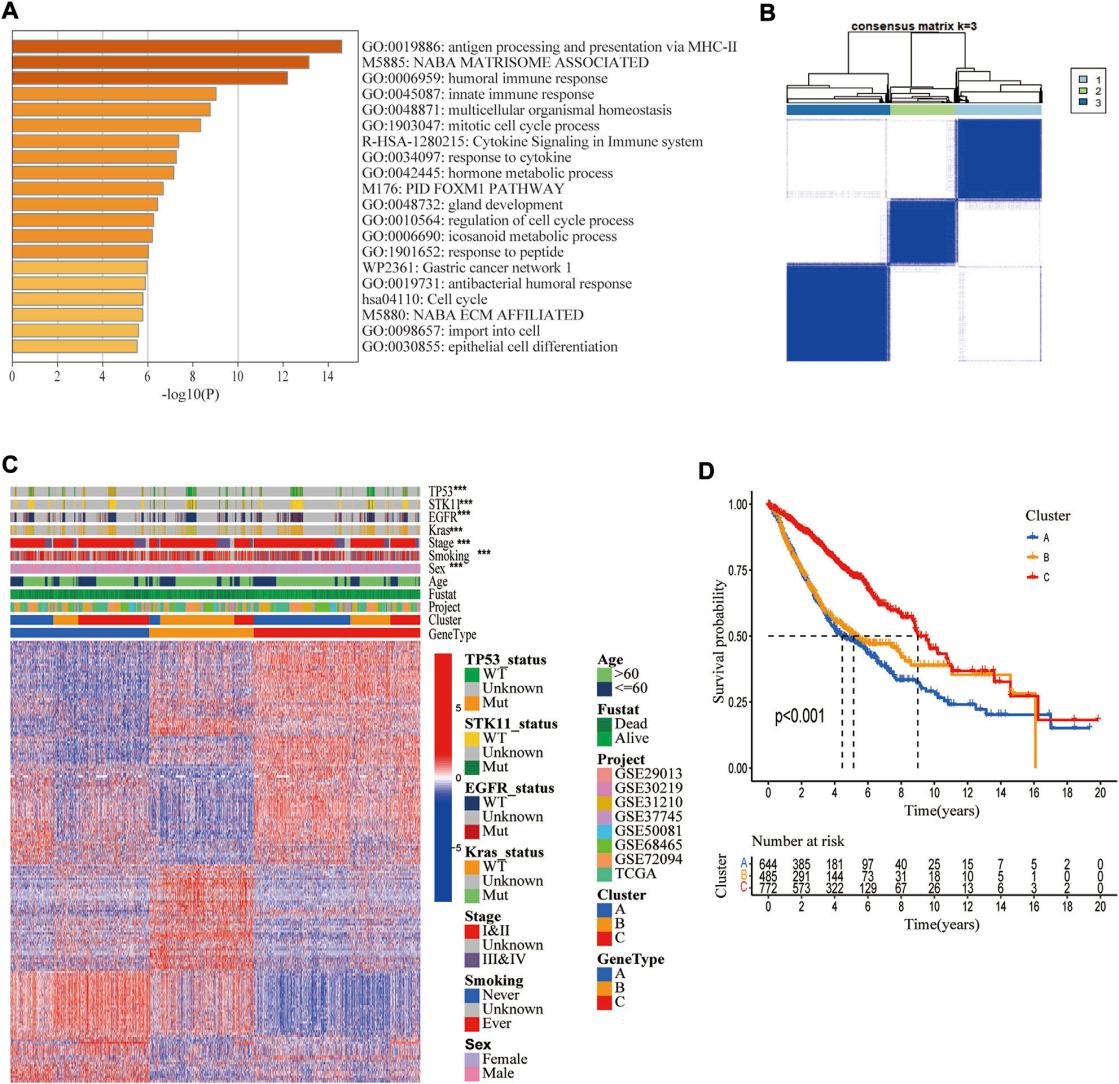
Identification of three cuproptosis-related genetic subtypes. **(A)** The enrichment of biological pathways based on differentially expressed genes (DEGs) in three cuproptosis molecular subtypes. **(B)** The 1972 LUAD samples in the merged cohort were classified into three genetic subtypes based on these DEGs using unsupervised clustering analysis (k = 3). **(C)** Heatmap showing differences in DEGs and different clinical features in three genetic subtypes. **(D)** Kaplan Meier Analysis showed differences in overall survival among three genetic subtypes.*, **, and ***, represent *p* < 0.05, *p* < 0.01, and *p* < 0.001, respectively.

To explore the biological activity of cuproptosis, we identified 194 DEGs associated with cuproptosis molecular subtypes using the “limma” R package. The functional enrichment analysis showed that these genes were significantly enriched in immune-related biological activities and metabolic pathways (Figure 5A). GO and KEGG analyses also showed significant enrichment in immunological and cancer-related pathways (Supplementary Figure S7), suggesting that cuproptosis is essential for immune control of TME (Figure 5B). The heatmap demonstrated a strong correlation between genotype A vs. P53 mutation, genotype B vs. STK11 mutation, advanced TNM stage vs. KRAS mutation, and genotype C vs. EGFR mutation (Figure 5C). Additionally, the Kaplan-Meier plot demonstrated that patients with genotype A had the worst overall survival while those with genotype C had the greatest OS (log-rank test, *p* < 0.001, Figure 5D).

### Construction of the cuproptosis signature

**FIGURE 6:**
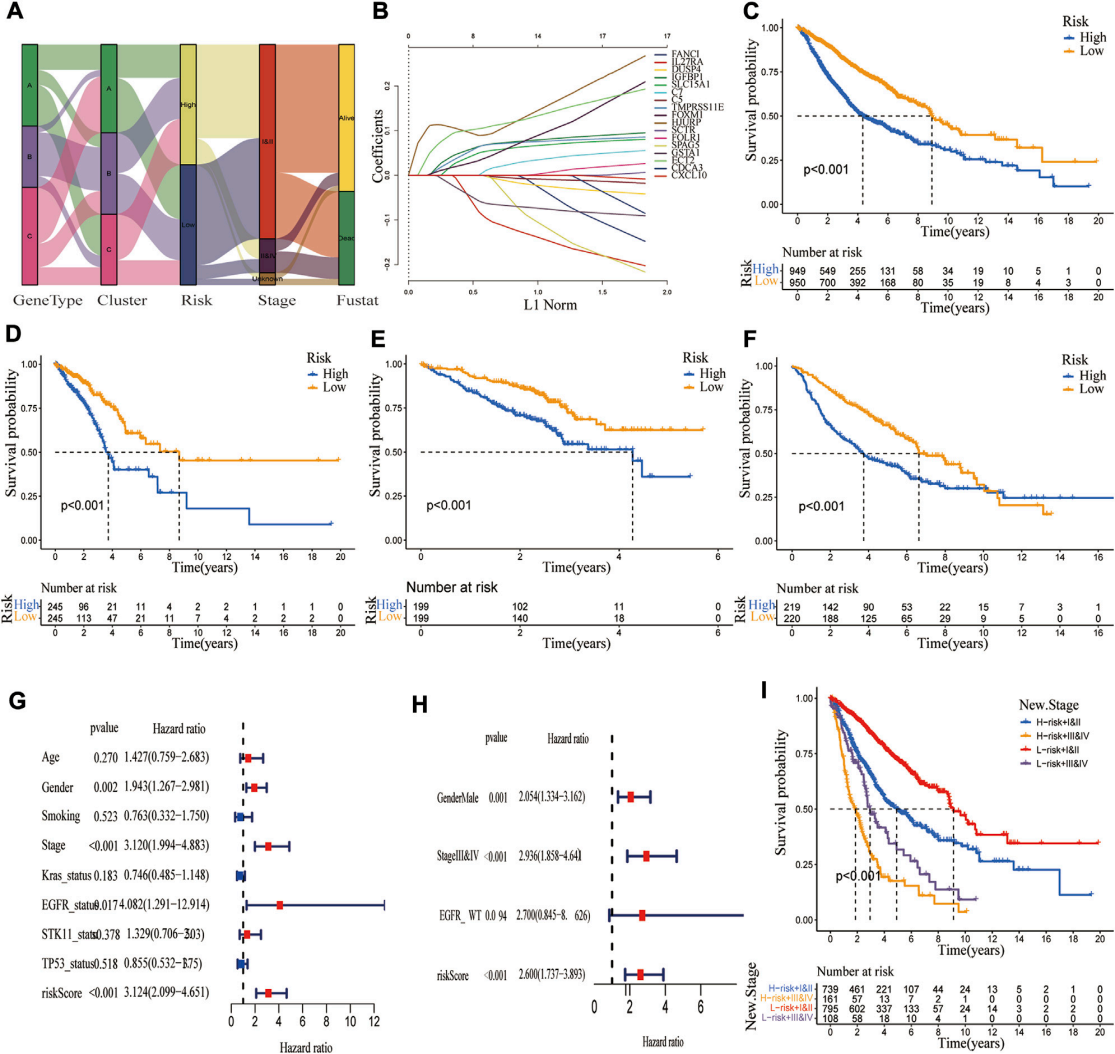
Construction of a CRG_score signature in the merged cohort and validation in three independent datasets. **(A)** The alluvial diagram showed the changes in LUAD molecular clusters, genetic subtypes, CRG_score, TNM stage, and survival status. **(B)** The Lasso regression analysis identified 17 LUAD prognosis-related genes to construct the CRG_score signature. The difference in overall survival between high and low CRG_score groups in **(C)** merged cohort; **(D)** TCGA; **(E)** GSE72094; **(F)** GSE68465 (*p* < 0.001). **(G,H)** Univariate and multivariate Cox regression analyses showed the prognostic value of the CRG_score in the merged cohort. **(I)** Kaplan Meier curve revealed the relationship among TNM stage, CRG_score, and survival probability.

A CRG_score signature was constructed based on 194 DEGs. Figure 6A depicts the distribution of patients in the three molecular subtypes, three genotypes, and two CRG_score groups. Univariate/multivariate Cox regression analysis yielded 17 prognostic genes (Supplementary Table S8). Using Lasso and multivariate Cox analyses, the CRG_score signature was constructed, with the risk coefficient for each gene shown in Supplementary Table S9. Finally, the above 17 genes were incorporated into our CRG_score signature (Figure 6B). The LUAD patients were classified as high-risk (*n* = 949) or low-risk groups (*n* = 950) based on the median risk score. Using Kaplan-Meier analysis, we discovered that patients in the high-risk group had a significantly worse OS than in the low-risk group (*p* < 0.001, Figure 6C). Given that TCGA-LUAD, GEO72094, and GEO68465 had relatively large LUAD samples and corresponding clinical data, they were used to assess the reliability of our CRG_score signature from four aspects, including OS difference, mortality risk identification, classification, and prognostic ability. These results indicated that our signature exhibited a good performance (Figures 6D–F; Supplementary Figure S8). Although it is widely acknowledged that the tumor TNM staging substantially influences patient survival, patients with same stage LUAD can have a considerably variable prognosis, which may be connected to lung cancer heterogeneity. We observed that combining our CRG_score with TNM staging may identify patients with a poor prognosis more accurately. These findings indicated that individuals with low-risk and stage I/II had the best prognosis, while those with high-risk stage and III/IV had the worst (Figure 6I).

### Cuproptosis-related gene_score was an independent risk factor, with the ability to predict the OS of lung adenocarcinoma patients

To explore the ability of the CRG_score signature to stratify clinical characteristics, we investigated the relationship between CRG_score and various clinical variables (age, gender, smoking status, TNM stage, EGFR status, BRAF status, KRAS status, STK11 status, and P53 status). As shown in Supplementary Figure S9, Kaplan-Meier curves showed that OS was significantly longer in the low-risk group than in the high-risk group patients with different ages (*p* < 0.01), sex (*p* < 0.001), smoking history (*p* < 0.001), TNM stage (I/II *p* < 0.001, III/IV *p* = 0.002 for stage III/IV), EGFR status (*p* < 0.001 for WT, *p* = 0.007 for Mut), Kras status (*p* < 0.001 for WT, *p* < 0.026 for Mut), STK11 status (*p* < 0.001 for WT, *p* = 0.879 for Mut), TP53 status (*p* < 0.001 for WT, *p* = 0.062 for Mut), indicating that our CRG_score exhibited good performance in patient stratification. To explore whether this CRG_score could independently predict OS, we performed univariate and multivariate Cox regression analyses combining their clinical characteristics and risk score. As shown in Figures 6G,H, the CRG_score was an independent risk factor (*p* < 0.001, HR: 3.124, 95% CI: 2.099-4.651). Similar results were obtained in three independent cohorts (Supplementary Figure S10).

### The characteristics of cuproptosis signature in the cancer genome atlas cohort

**FIGURE 7:**
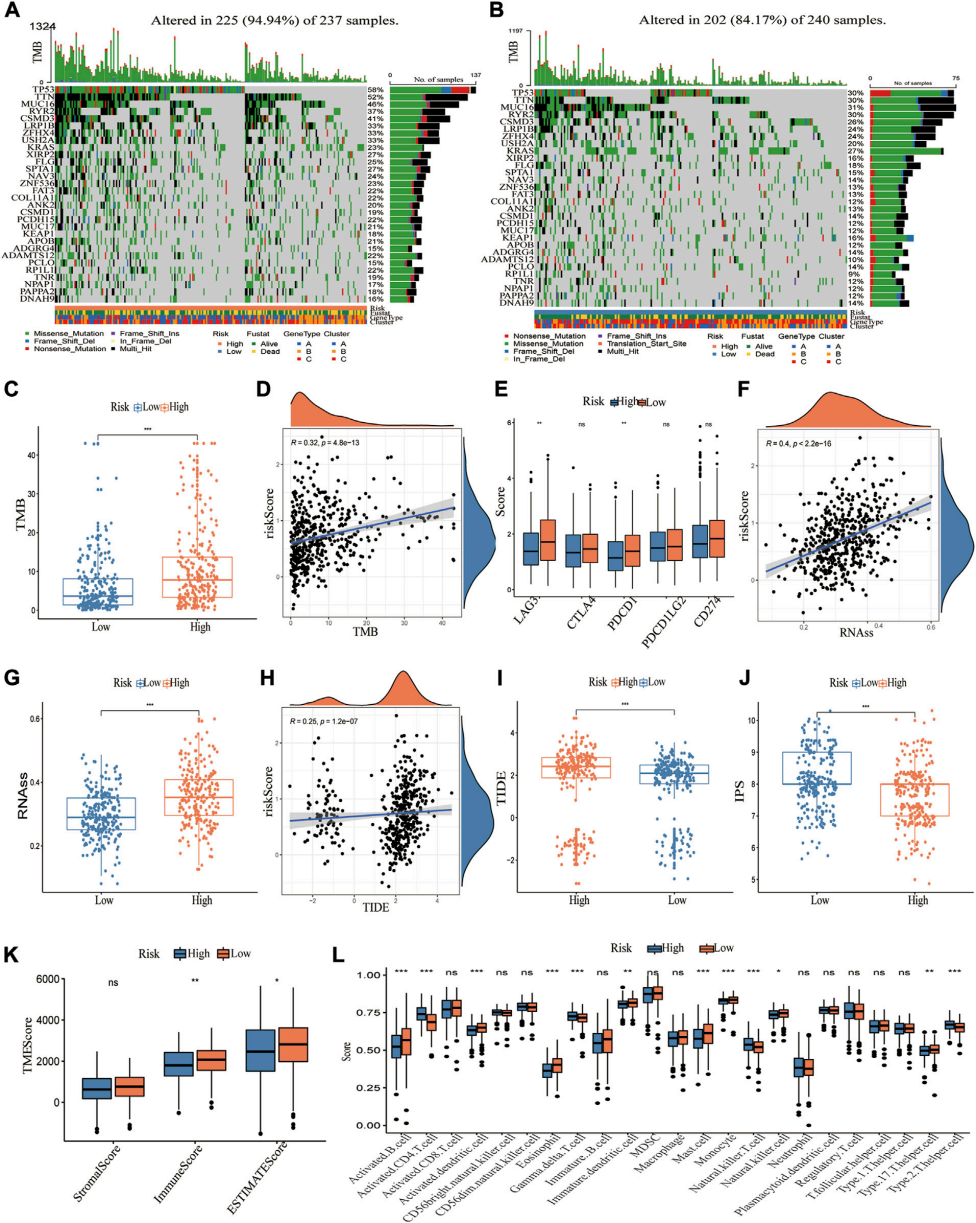
Characteristics of CRG_score in TCGA cohort and predicting immunotherapy response. **(A,B)** The waterfall plot depicts the heterogeneity in the somatic mutation landscape of tumors across groups with low and high CRG_score. **(C,D)** Significant differences of TMB in high and low CRG_score groups and correlation with the risk score. **(E)** Differential expression of immune checkpoint-related genes in high and low CRG_score groups. **(F,G)** Significant differences of RNAss (cancer stemness cells index) in high and low CRG_score groups and correlation with the risk score. **(H,I)** Significant differences of TIED score in high and low CRG_score groups and correlation with the risk score. **(J)** The boxplot demonstrated that IPS was significantly higher in the high CRG_score group than in the low CRG_score group. **(K)** The TME score in the high and low CRG_score group. (i) 23 TME cells infiltration for the two risk groups. *, **, and ***, represent *p* < 0.05, *p* < 0.01, and *p* < 0.001, respectively.

The somatic mutations were compared in high and low CRG_score groups, and the top 30 genes with the greatest mutation frequency were identified (Figures 7A,B). It was observed that the high CRG_score had higher mutation frequencies, such as TP53 and KRAS mutations, strongly associated with cancer. The high CRG_score group displayed a greater tumor mutation burden (TMB) than the low CRG_score group (Figures 7C,D). Then, the correlation between these risk groups and immune checkpoint-related genes was explored. PD-1 and LAG3 expressions were significantly greater in the low CRG score group, but PD-L1 (CD274), CTL4, and PD-2 (PDCD1LG2) expressions showed no difference (Figure 7E). The CSC index was significantly higher in the high-risk group than in the low-risk group (Figure 7F). A positive association was found between CRG_score and CSC index (R = 0.4, *p* < 0.001, Figure 7G), indicating that LUAD patients with higher CRG_score exhibited more evident stem cell features and reduced cell differentiation. Considering the important role of CRGs in tumor immunity and tumor microenvironment, we further explored the relationship between CRG_score and immunotherapy. As a predictor of immunotherapy response, the IPS exhibited a better performance in identifying patients who benefit from immunotherapy. The IPS was significantly higher in the low CRG_score group than in the high CRG_score group (Figures 7G,H). Lower TIDE scores, T-cell exclusion scores, and higher T-cell dysfunction scores were associated with better responses to anti-PD-1 and anti-CTLA-4 immune checkpoint blockers (ICBs) (Jiang et al., 2018). TIDE and T-cell exclusion scores were significantly higher in the high CRG_score group, While the T-cell dysfunction scores were lower (Figures 7I,J; Supplementary Figures S11D–G). Given that TME can also influence immunotherapy, we examined immune cell infiltration in distinct CRG_score groups. The results indicated that the low-risk group had higher immune and ESTIMATE scores (Figure 7K) and greater infiltration of the majority of immune cells (Figure 7L; Supplementary Figures 10A–C). Interestingly, the clinically widely used drugs for LUAD, such as Paclitaxel, Docetaxel, Cisplatin, and Gefitinib, had a lower IC50 in the high-risk group, implying that the high-risk group was more sensitive to these drugs (Supplementary Figure S12).

### Relationship between the cuproptosis-related gene_score and the effect of immunotherapy in the IMvigor210 cohort

**FIGURE 8:**
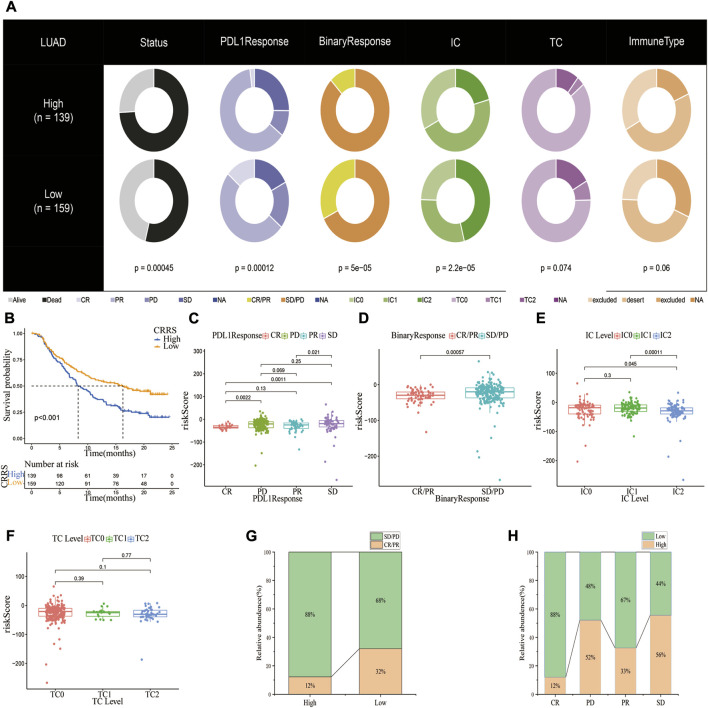
The potential of CRG_score signature in anti-PD-1/L1 immunotherapy. **(A)** Distribution of clinical characteristics among high and low CRG_score groups in the IMvigor210 cohort. **(B)** Kaplan-Meier plot showing the difference in overall survival in different CRG_score subgroups. **(C,D)**The risk scores in patients with CR/PR or CD/SD (PR, Partial Response, PD, Progressive Disease; SD, Stable Disease, and CR, Complete Response). In the IMvigor210 cohort, the difference in CRG_score between PD-L1 expression of various **(E)** IC and **(F)** TC. **(G)** Proportions of anti-PD-L1 immunotherapy response in different CRG_score groups. **(H)** The proportion of patients having a high or low-risk score in various responses to anti-PD-L1 immunotherapy.

The value of the CRG_score in predicting response to immunotherapy was further studied in an anti-PD1/PD-L1 treatment cohort (IMvigor210). Patients were classified into high and low CRG_score subgroups based on the optimal CRG_score cut-off value. Figure 8A depicts the clinical features of the IMvigor210 data. Patients with a low CRG_score had a better prognosis (Figure 8B). Additionally, there was a higher risk score in the SD/PD group compared to the CR/PR group (Figures 8C,D). Next, the association between the CRG_score and the tumor-infiltrating immune cells (IC) and tumor cells (TC) immune types was examined. The CRG score was substantially lower in IC2 than in IC0 or IC1, but no significant difference was observed across TC immune types (If the IC was <1%, ≥1% but <5%, ≥5% but < 10%, or ≥10% in PD-L1 positive patients, the specimens were scored as IHC IC0, IC1, IC2, or IC3, respectively. In PD-L1 positive patients, if TC in the specimens was <1%, ≥1% but <5%, ≥5% but <50%, or ≥50%, they were graded as IHC TC0, TC1, TC2, or TC3, respectively) (Figures 8E,F). Compared to the high-risk subgroup, the low-risk subgroup exhibited an almost threefold higher response rate (complete response or partial response) (32% vs. 12%) and a lower SD/PD (PD, Progressive Disease; SD, Stable Disease) rate (Figures 8G,H). Taken together, our findings implied that the CRG_score could be a reliable predictor of immunotherapy response.

### The construction of a nomogram for predicting OS

**FIGURE 9:**
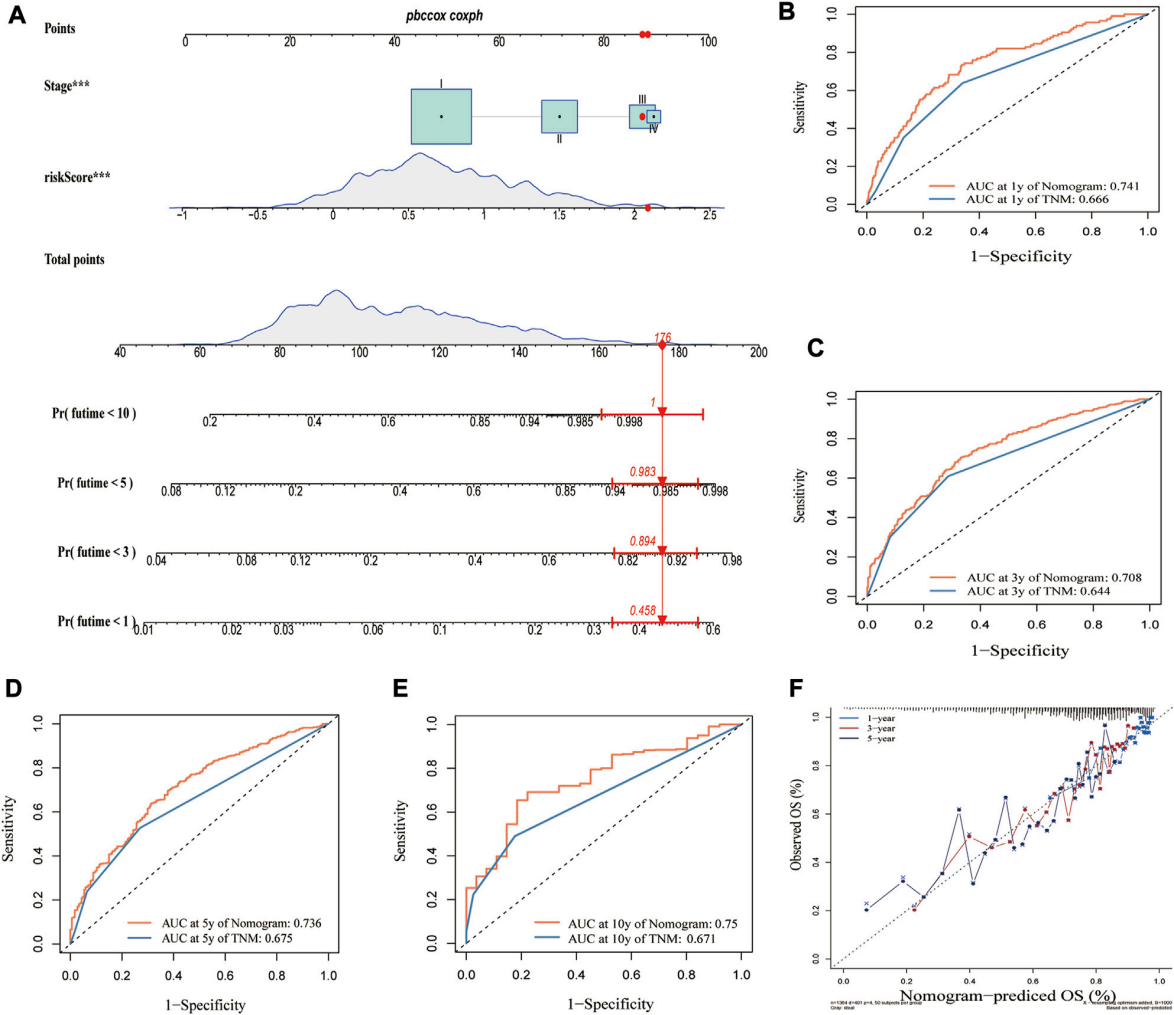
Construction of a nomogram based on CRG_score and TMN stage. **(A)** Nomogram for predicting the 1-, 3-, 5-, and 10-year OS of LUAD patients in the merged cohort. Comparing AUCs for the nomogram and TNM stage in predicting OS at **(B)** 1 year, **(C)** 3 years, **(D)** 5 years, and **(E)** 10 years in the merged cohort. **(F)** Calibration plot of the nomogram for predicting the probability of the 1-, 3-, and 5-year OS.

Given that we established the clinical utility of the CRG_score in predicting the survival of LUAD patients, we established a nomogram incorporating the CRG_score and TNM staging to predict the survival of LUAD patients at 1, 3, 5, and 10 years (Figure 9A). When we compared the predictive accuracy of our nomogram with TNM staging, the nomogram showed AUC values of 0.741, 0.708, 0.736, and 0.75 for 1, 3, 5, and 10 years, respectively, compared with 0.666, 0.644, 0.675, and 0.671 for TNM staging (Figures 9B–E). The calibration plot showed excellent agreement between our nomogram and actual observations regarding 1-, 3-, and 5-year survival probability (Figure 9F).

## Discussion

Herein, we examined the transcriptional and genetic alternation of CRGs. We discovered potential co-transcription factor motifs and sensitivity drugs that target these differentially expressed CRGs. Additionally, we grouped LUAD into three molecular subtypes (Clusters A–C) based on the expression of 16 CRGs, with Cluster A having the best prognosis. Further analysis revealed substantial differences in immune cell infiltration and TME. Significant heterogeneity was observed between the three molecular subtypes in cancer-related signaling pathways, including T cell co-stimulation-induced inflammation-promoting pathways, MYC targets v1, MYC targets v2, the G2M checkpoint, and E2F. Based on the expression of DEGs, we identified three genotypes and constructed a CRG_score signature. This signature demonstrated excellent stratification and predictive potential as an independent risk factor for LUAD. The different risk subtypes showed significant differences in prognosis, somatic mutations, TMB, CSC index, TIDE score, T-cell exclusion score, T-cell dysfunction score, TME, immune checkpoints, and drug sensitivity. Furthermore, the CRG_score was a reliable predictor for immunotherapy response, which was corroborated in IMvigor210. Finally, we established a predictive model by combining the CRG_score and TNM staging, which could reliably predict the OS of LUAD at 1, 3, 5, and 10 years. To summarize, our findings provide novel insights into the molecular mechanisms driving LUAD.

Programmed cell death plays a fundamental role in various pathological and physiologic processes, including cancer (Fuchs and Steller, 2011). Well-established PCD forms include apoptosis, necroptosis, pyroptosis, and ferroptosis (Pan et al., 2022a), which play a key role in tumor immunity and treatment strategies (Carneiro and El-Deiry, 2020; Tang et al., 2020). The relationship between PCDs regulators and immunological markers has also been investigated in several cancers, including LUAD (Zhang et al., 2021b; Pan et al., 2022b; Zou et al., 2022). In this study, we predicted the phenotype, treatment response, and prognosis by clustering tumor patients based on their molecular signatures, such as ferroptosis-related gene signatures. Nonetheless, the exact role of cuproptosis in LUAD remains largely unknown.

Moreover, the CRGs-based signature yielded good performance in predicting prognosis and immunotherapy response in our research. Therefore, cuproptosis can potentially participate in the development of LUAD.

Current evidence suggests that cuproptosis depends on the direct binding of copper to the thioctylated proteins in TCA, which induces abnormal oligomerization of thioctylated proteins. In addition, copper reduces Fe-S cluster protein levels. These two processes subsequently induce a proteotoxic stress response and a distinct form of cell death (Tsvetkov et al., 2022). Cuproptosis can reportedly be rescued by knocking down CRGs, such as FDX1, LIPT1, LIAS, DLD, DLAT, PDHA1, and PDHB(8). FDX1, DLAT, and LIAS are widely acknowledged to be essential for inducing cuproptosis. Ferredoxin 1 (FDX1) converts divalent copper ions to the more deleterious monovalent copper ions while regulating protein lipoylation. FDX1 knockdown has been associated with protein lipoylation deficiency of DLAT and decreased cellular respiration, consistent with the results of LIAS knockdown (Tsvetkov et al., 2022). Zeyu Zhang et al. found that knocking down FDX1 in A549 cells did not suppress tumor cell growth or cause apoptosis but changed cell metabolism (Zhang et al., 2021a), which may be attributed to reduced cuproptosis in A549 cells after knocking down FDX1. In another study, FDX1 was significantly associated with immune infiltration levels and programmed cell death protein 1 (PD-1) expression in clear cell renal cell carcinoma (Bian et al., 2022). In the present study, FDX1 was lowly expressed, suggesting it is a favorable prognostic factor. We hypothesized that upregulation of the FDX1 gene leads to protein lipoylation and subsequently disrupts mitochondrial respiration. It may inhibit the proliferation of lung adenocarcinoma cells, thereby suppressing tumor growth. Haozhen Lv et al. demonstrated that LIPT1 could predict prognosis and revealed a strong correlation between LIPT1 expression and immune infiltration in melanoma (Lv et al., 2022). In our study, upregulated LIPT1 expression correlated with poor outcomes of LUAD. Therefore, we speculate that upregulation of LIPT1 may inhibit tumorigenesis and progression by disrupting TCA in mitochondria, thereby triggering cuproptosis. Indeed, additional research is required to confirm these hypotheses.

Lung carcinogenesis involves a series of complex processes involving intrinsic genetic abnormalities in tumor tissue and the tumor’s interaction with immune cells in the surrounding TME. It has been established that TME has a major effect on tumor growth, progression, and resistance to treatment (Remark et al., 2015; Hinshaw and Shevde, 2019). CD4^+^ Th1 cells activated CD8^+^ T cells, and γδ-T cells are typically implicated in type I immune responses and are associated with a favorable prognosis in patients with lung cancer (Schalper et al., 2015; Bremnes et al., 2016). On the other hand, Th2, Th17, and Foxp3+ regulatory T (Treg) cells are generally related to tumor development and poor prognosis (Marshall et al., 2016). Tumor-infiltrating B lymphocytes (TIBs) have been found to produce an effective and favorable immune response in the majority of solid tumors (Sautes-Fridman et al., 2016). Petitprez et al. (Petitprez et al., 2020) concluded that B-cell enrichment was the strongest predictor of prolonged survival in soft tissue sarcomas. In this study, significant changes in TME features and tumor-infiltrating immune cells (TIICs) were observed across the three molecular subtypes and different CRG_score subgroups. B cells, TIL, CD4^+^ T cells, activated dendritic cells, and neutrophils were significantly enriched in cluster A and the low CRG_score group, which had the best prognosis. However, cluster B and the high CRG_score group had a poorer prognosis due to immunosuppression. These results imply that CRGs play a vital role in the cancer immunity of LUAD.

ICBs have broadened the therapeutic landscape for advanced lung cancer patients and represent a standard frontline strategy for monotherapy or in combination with other therapies. Moreover, it has become an option in patients with oncogene-addicted non-small cell lung cancer (NSCLC) following the failure of targeted therapies. However, predictive indicators are urgently needed, as ICBs are effective in only a minority of patients, while substantial immunotoxicity side effects are possible. The TIDE score, which incorporates both mechanisms of T cell dysfunction (T cells dysfunction score) and T cell immune exclusion (T cells exclusion score) in tumors, is a better predictor of immunotherapy response than TMB and PD1/PD-L1 (43). A lower TIDE score shows that tumors are more susceptible to anti-PD-1/PD-L1 and anti-CTLA4 ICBs (Jiang et al., 2018). The TIDE and T cells dysfunction scores were positively connected with the CRG_score in our study, showing that the low-risk group was more sensitive to immunotherapy, consistent with the IPS results. We further analyzed the relationship between CRG_score and immunotherapy efficacy in the Imvigor210 cohort, which validated our conclusion that the low CRG_score group was more likely to benefit from immunotherapy. However, our study found that only PD-1 levels were significantly greater in the low CRG_score group than in the high CRG_score group, whereas PD-L1 and CTLA4 levels were comparable. These findings suggest that PD-L1 or CTLA4 are not accurate predictors of immunotherapy response. The KEYNOTE-189 study demonstrated that patients with low PD-L1 expression could benefit from immunotherapy (Gadgeel et al., 2020). In the latest KEYNOTE-091 study, pembrolizumab significantly improved disease-free survival (DFS) for lung cancer regardless of PD-L1 expression levels. In contrast, for the PD-L1 high expression population (TPS ≥50%) treated with pembrolizumab, the DFS improved compared to the placebo group, but there was no statistically significant difference. Accordingly, our CRG score may replace this void in terms of immunotherapy response prediction.

It has been established that the higher the CSC index, the less differentiated the tumor is (Malta et al., 2018). In our study, we discovered that the CSC index was significantly higher in the high CRG_score group, and the IC50 values of Paclitaxel, Docetaxel, Cisplatin, and Gefitinib were significantly lower than in the low CRG_score group, indicating that the high CRG_score group was more susceptible to chemotherapy and targeted therapy.

Notwithstanding that our CRG_score has good classification and predictive potential, this research has some limitations. First, the enrolled cohorts analyzed were retrospectively collected. Accordingly, large prospective clinical studies and further *in vivo* and *in vitro* experimental studies are warranted. Besides, many key clinical variables were not assessed, including surgery, neoadjuvant chemotherapy, and radiation, which may influence immunotherapy and cuproptosis subtype prognosis.

In summary, we comprehensively investigated CRGs to reveal their possible role in TME, prognosis, and sensitive drugs of LUAD. These findings provide the foothold for accurate prognostic prediction and novel therapeutic strategies, especially for personalized treatment of this particular patient population.

## Data Availability

The original contributions presented in the study are included in the article/Supplementary Material, further inquiries can be directed to the corresponding author.

## Abbreviations

CRGs, cuproptosis-related genes; LUAD, lung adenocarcinoma; TME, tumor microenvironment; CRG_score, cuproptosis-related gene score; TIDE, tumor immune dysfunction and exclusion; ICB, Immune checkpoint blockade; IPS, immunophenoscore; CSC, cancer stem cell; IC50, half-maximal inhibitory concentration; LC, Lung cancer; SCLC, small cell lung cancer; NSCLC, non-small cell lung cancer; TCA, tricarboxylic acid cycle; DEGs, differentially expressed genes; OS, overall survival; GEO, Gene Expression Omnibus; TCGA, The Cancer Genome Atlas; FPKM, fragments per kilobase million; TPM, transcripts per kilobase million; CNV, copy number variation; GO, Gene Ontology; KEGG, Kyoto Encyclopedia of Genes and Genomes; TIICs, tumor-infiltrating immune cells
