# Supplementary material for: Identification of cuproptosis-related subtypes in lung adenocarcinoma and its potential significance
Source: Front Pharmacol. 2022 Oct 3;13:934722. doi: 10.3389/fphar.2022.934722 (PMC9573969; doi:10.3389/fphar.2022.934722)
Supplement: Supplementary file 1 [file DataSheet1.doc]

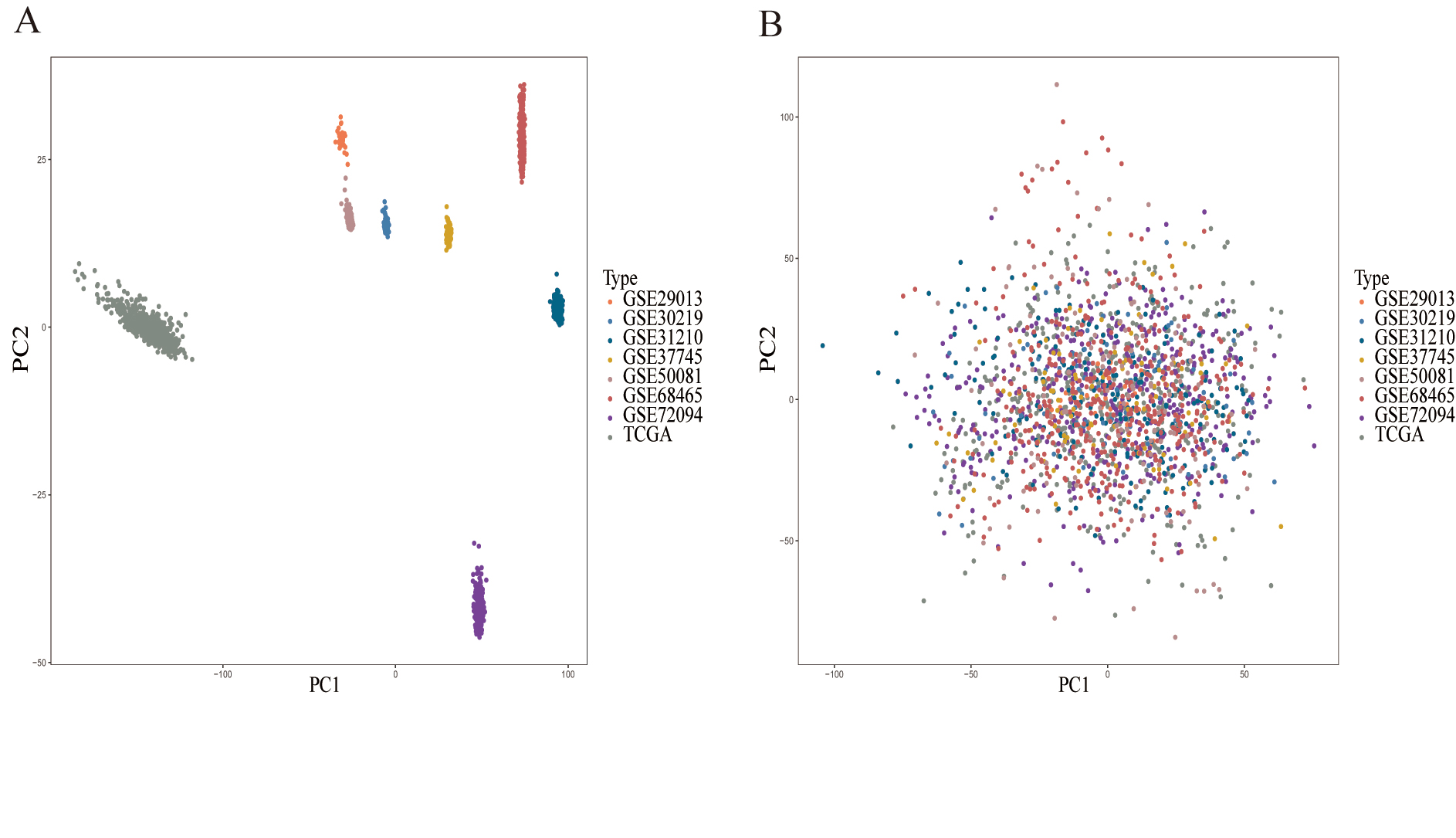


**Figure S1.** The batch effect before and after the combination. (A) GSE29013, GSE30219, GSE31210, GSE37745, GSE50081, GSE6846, GSE72094 and TCGA-LUAD have significant batch effect before combination. (B) The batch effect was eliminated in the merged cohort.


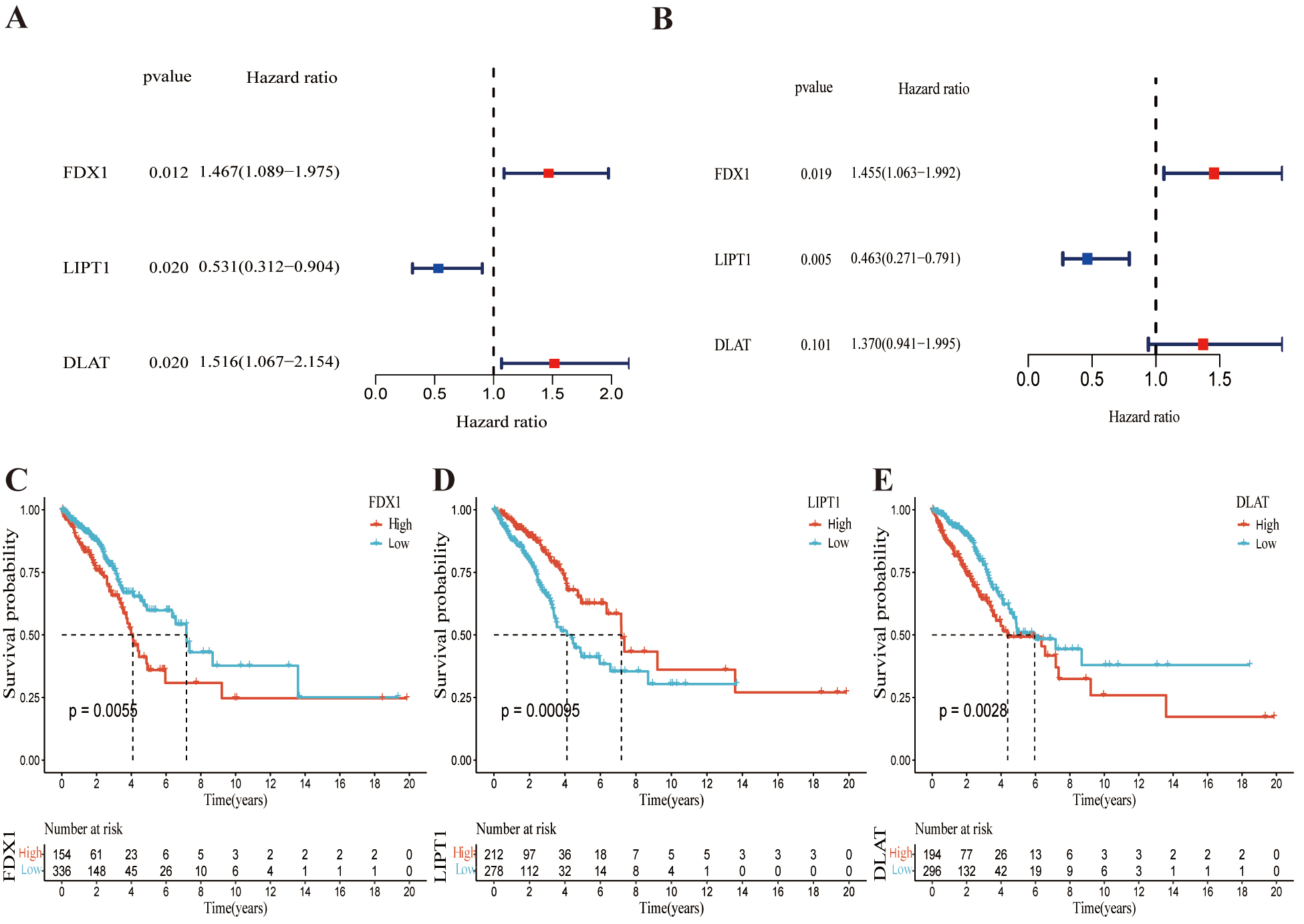


**Figure S2**.The prognostic value of cuproptosis-related genes. (A-B) Forest plot demonstrated cuproptosis-related genes with OS in LUAD by univariate/multivariate Cox regression analysis. (C-E) Kaplan–Meier analysis of FDX1, LIPT1, and DLAT in TCGA cohort, respectively. Log-rank P-value < 0.05 was considered significant (High, high expression level; Low, low expression level).


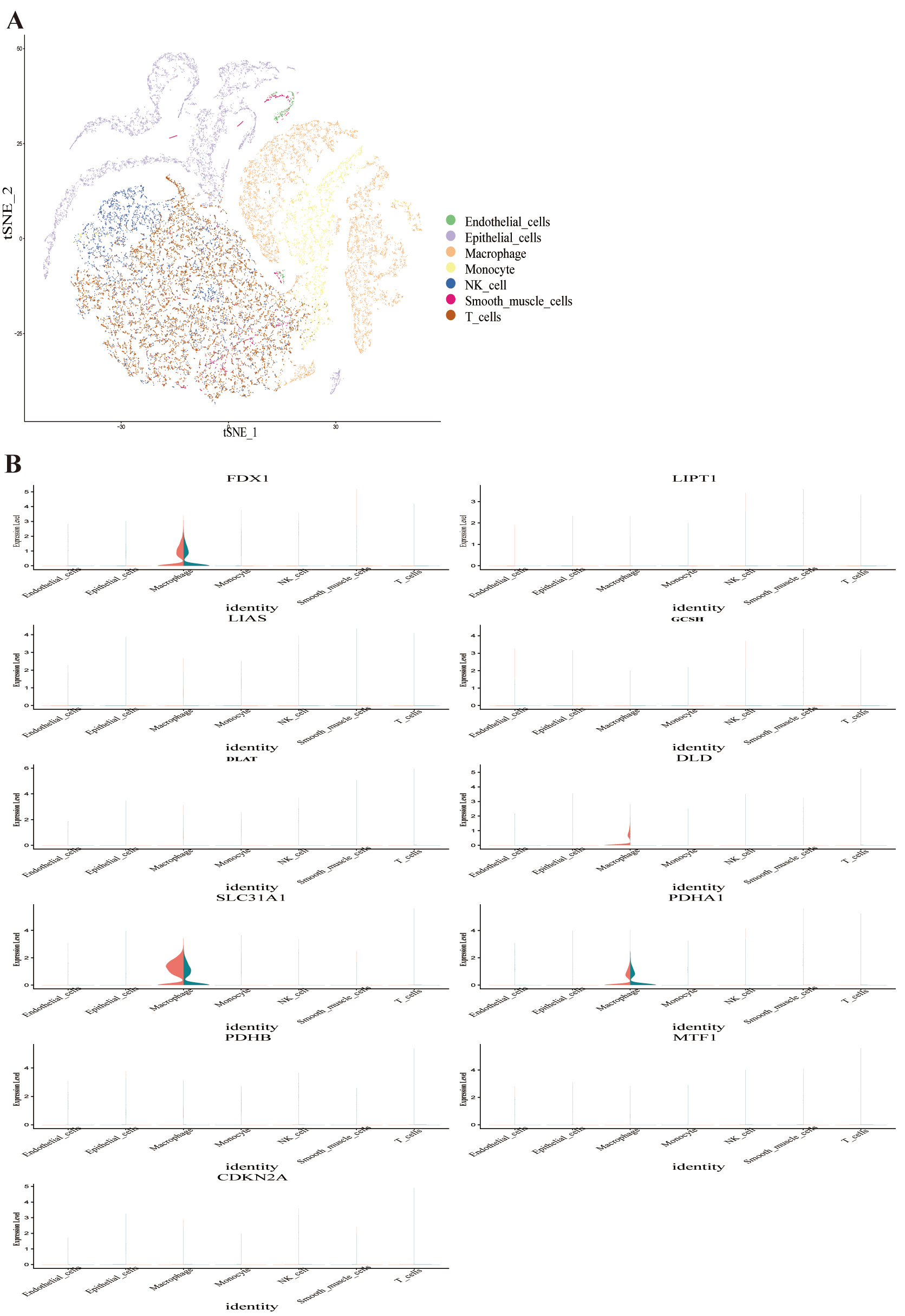


**Figure S3**. Cell-specific expression of differentially expressed cuproptosis-associated genes in our single-cell RNA-seq set, including three lung adenocarcinomas and three normal samples.(A)Lung tissue cells can be divided into seven main types.(B) FDX1,SXC31A1, DLD and PDHA1 were specificly expressed in macrophages(green: LUAD, red:normal tissue)


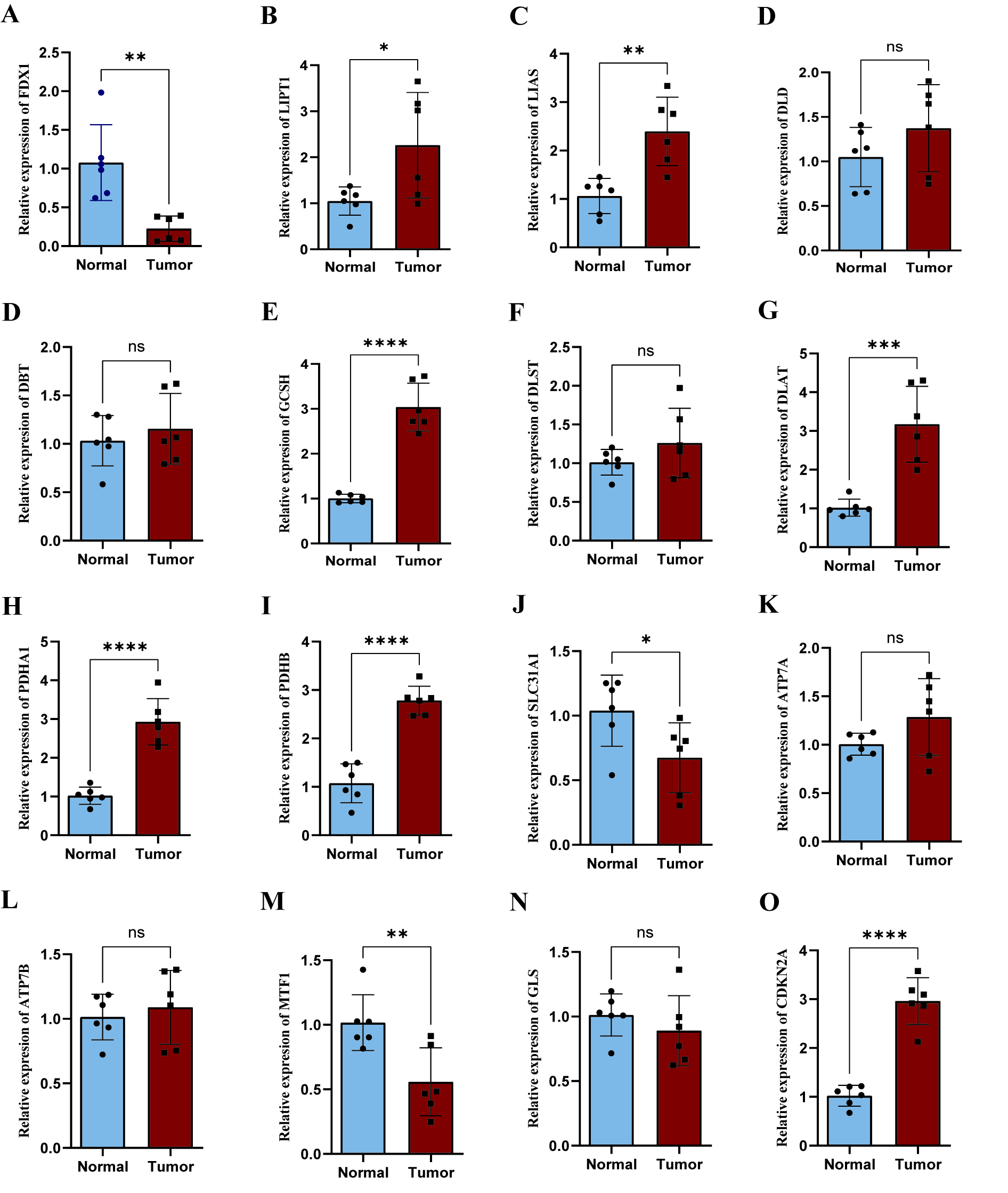


**Figure S4**. Expression levels of 16 cuproptosis-related genes in lung cancer tissues and corresponding normal tissues by RT-PCR.


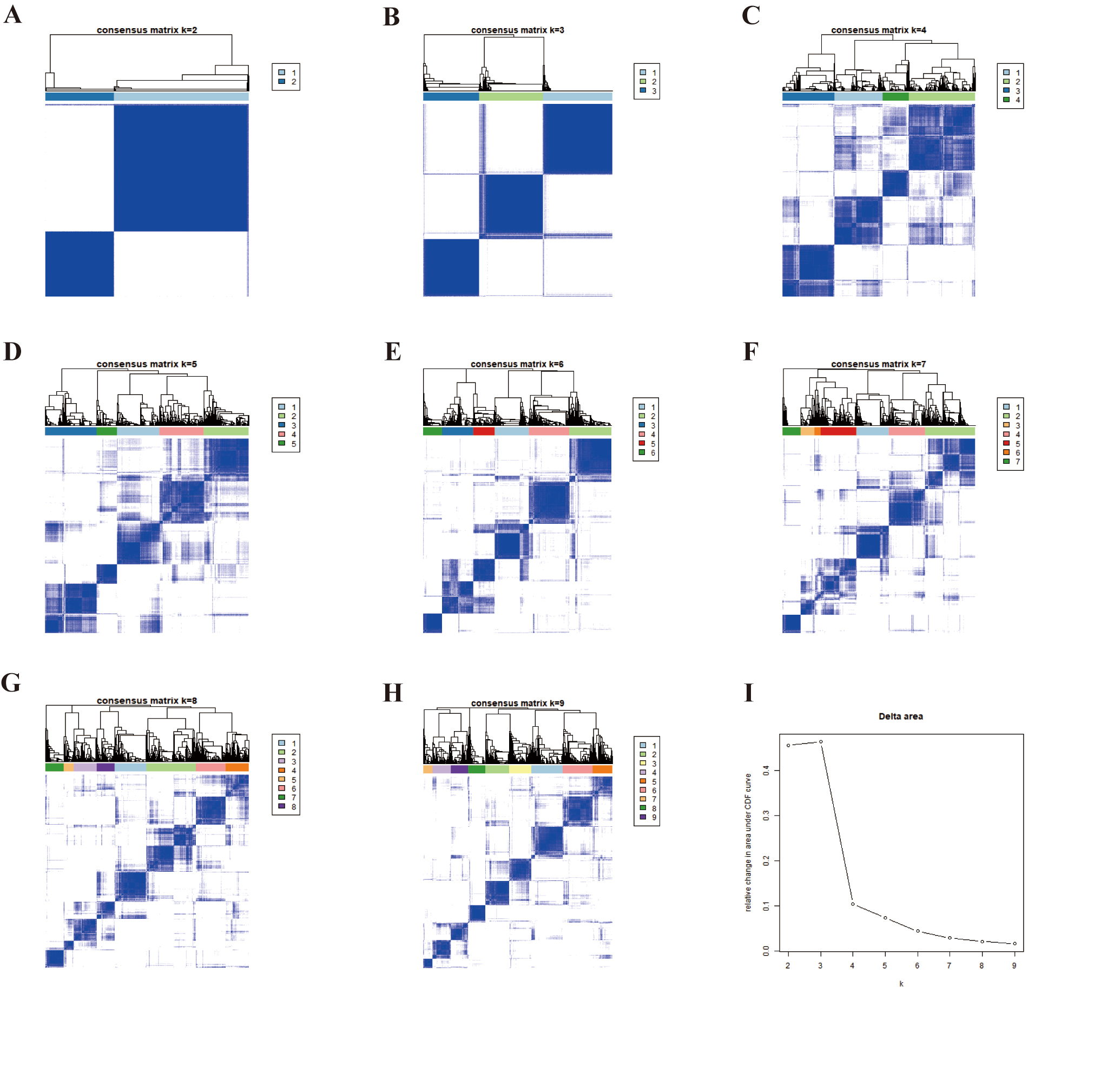


Figure S5. Unsupervised clustering of cuproptosis-related genes and Consensus matrix heatmaps for k = 2-9.


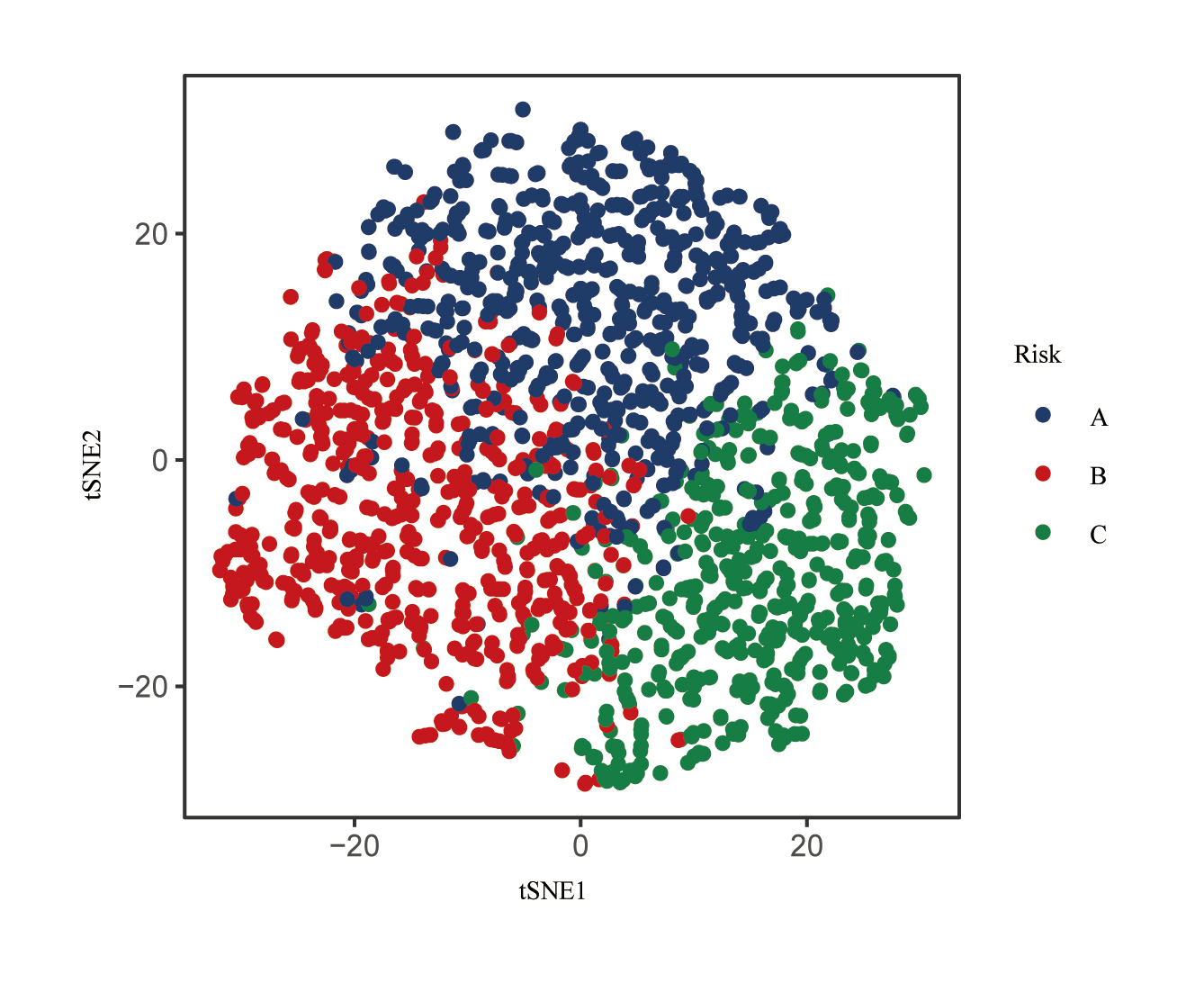


**Figure S6**. t-distributed stochastic neighbor embedding (t-SNE) analysis showed a remarkable difference in transcriptomes among cuproptosis molecular subtypes.


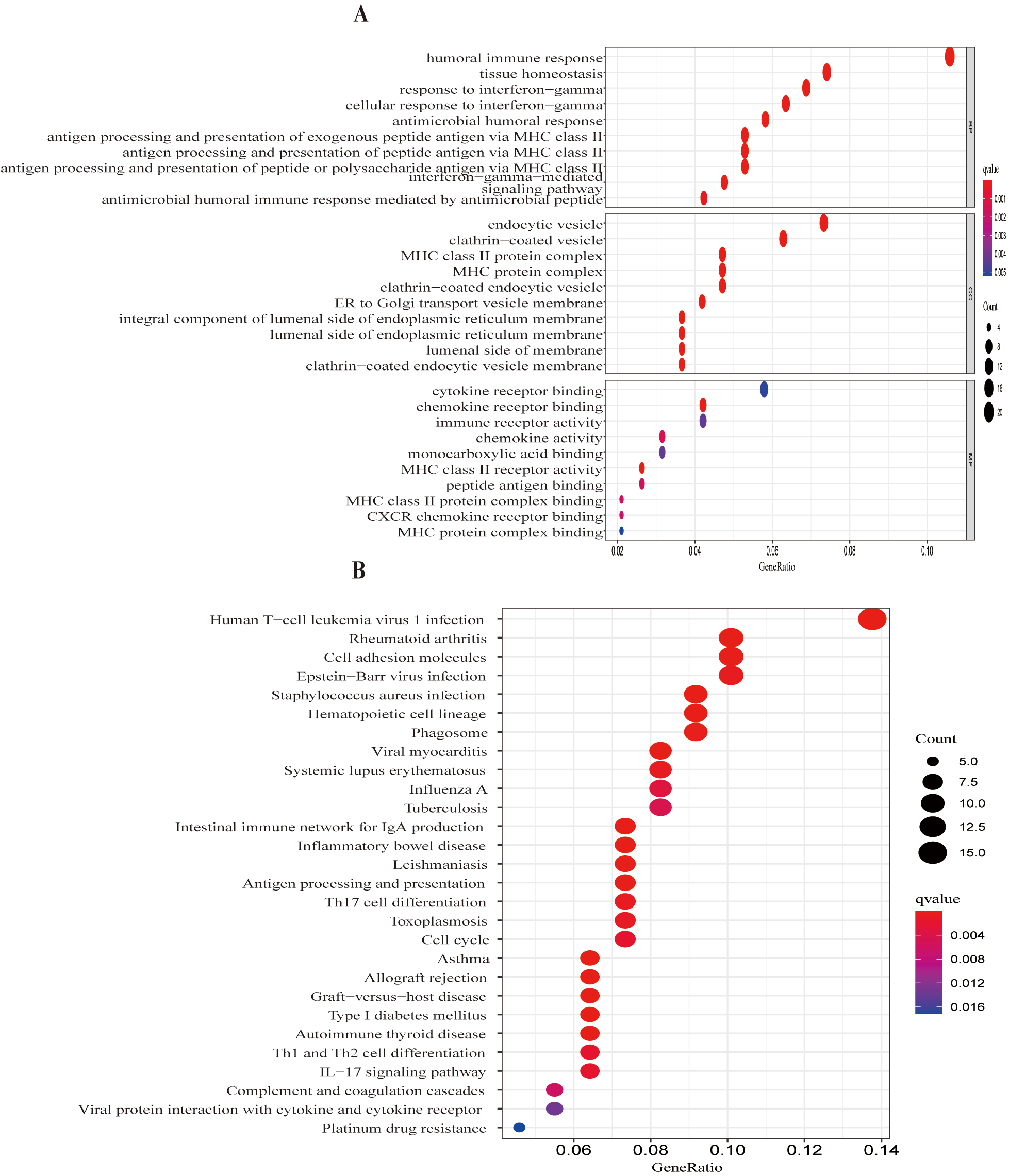


**Figure S7.** Enriched biological pathways in the three cuproptosis molecular subtypes. (A) GO analysis; (B) KEGG analysis


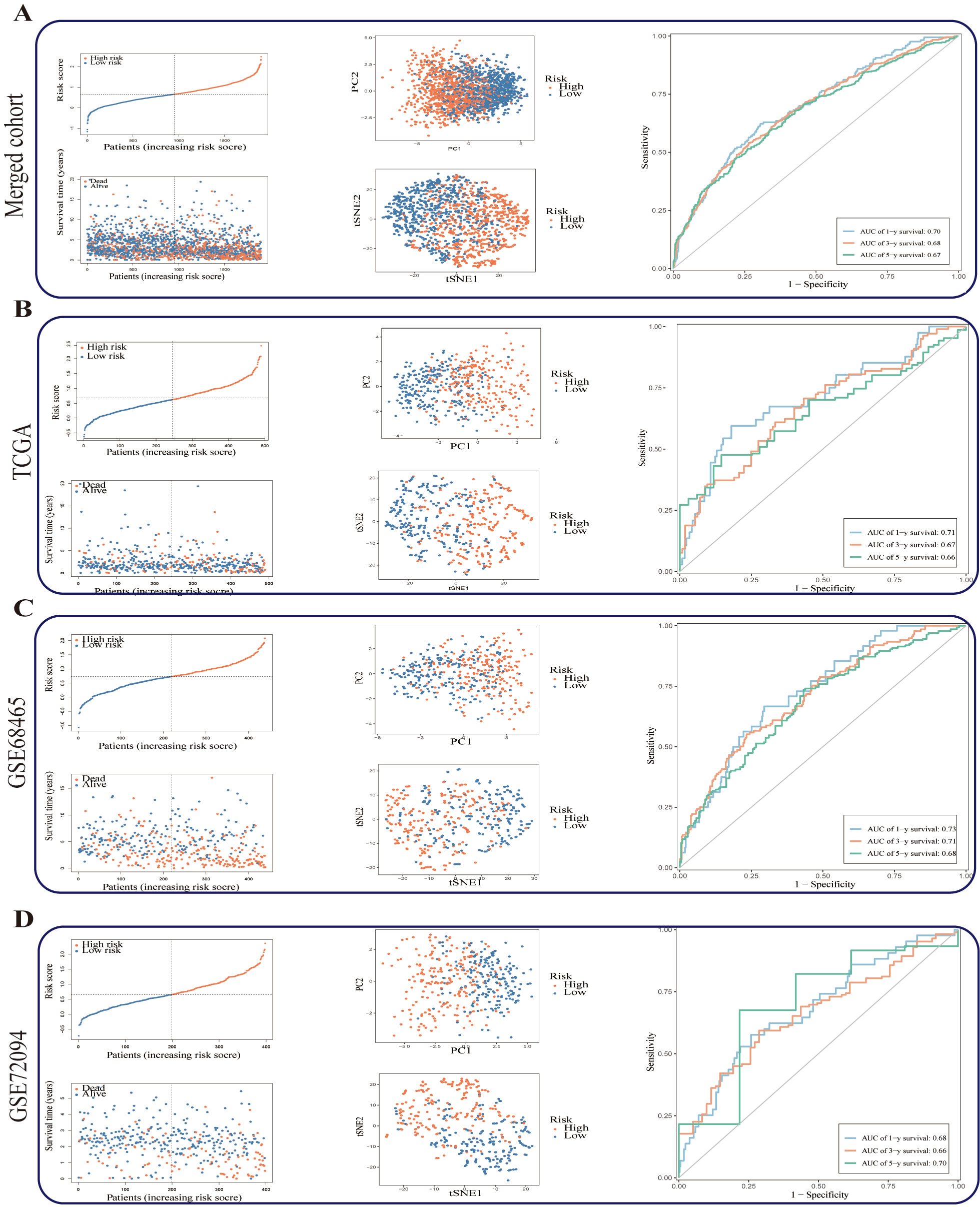


**Figure S8**.The reliability of the CRG_score signature was validated from three aspects including mortality mortality risk identification, classification by using PCA/tSNE analysis, and the prognostic ability for predicting 1-,3-,5- year survival in (A)Merged cohort; (B)TCGA; (C)GSE68468; (D)GSE72094.


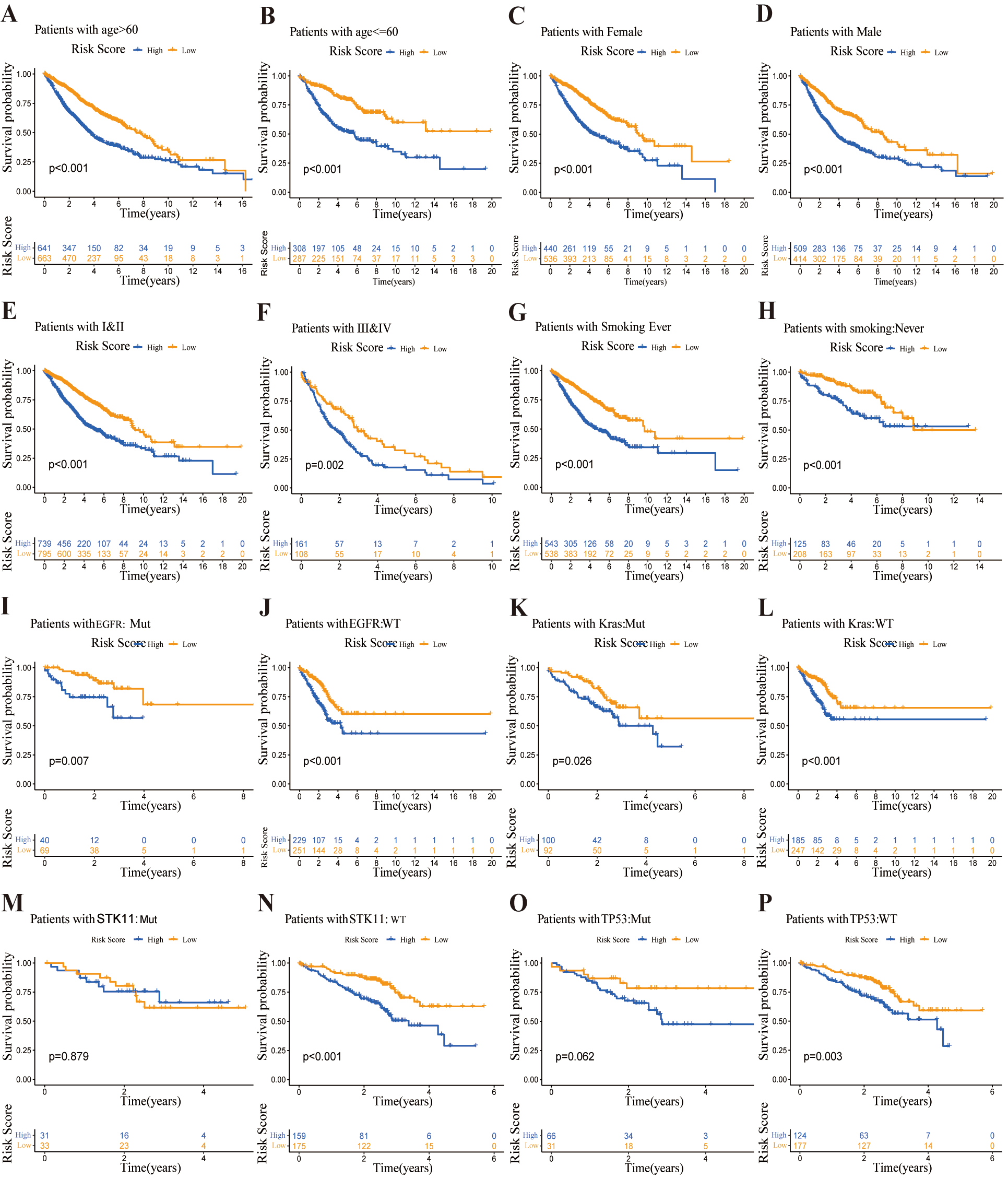


**Figure S9.** Stratification analysis of the CRG_score in LUAD.(A-B) Age ( age > 60 and age <= 60 years old). (C-D)Sex (female and male). (E-F) Tumor stage (I&II or III&IV). (G-H) Smoking history (ever or never). (I-J)EGFR status (Mut and WT). (K-L) KRAS status (Mut and WT).(M-N) STK11 status (Mut and WT).(O-P)TP53 status (Mut and WT).


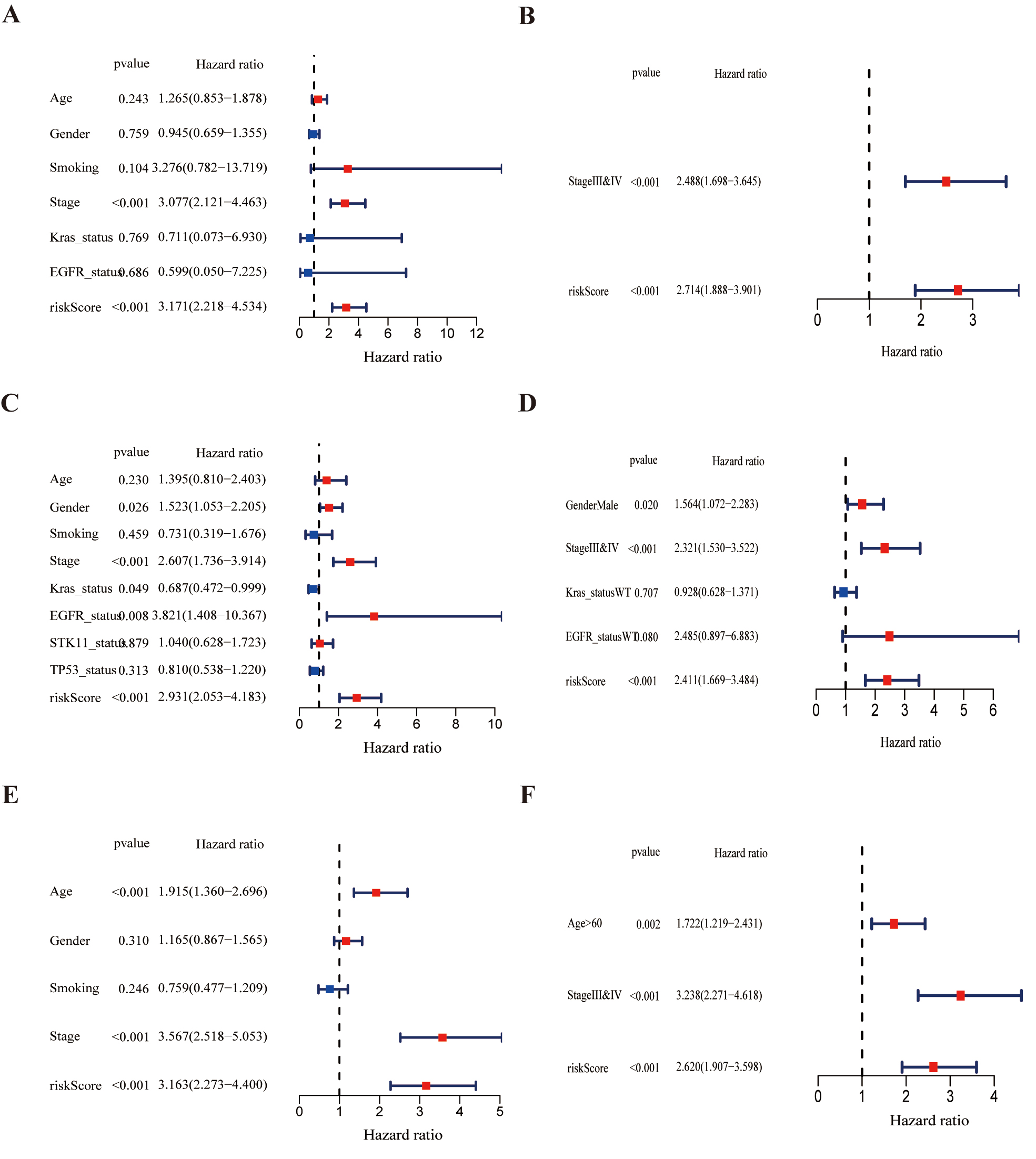


**Figure S10.** The correlation and independent prognosis analysis of CRG_score and clinicopathological variables in LUAD. Univariate and multivariate Cox regression analyses showed the prognostic value of the CRG_score in (A-B) the TCGA set; (C-D) GSE72094; (E-F)GSE68465.


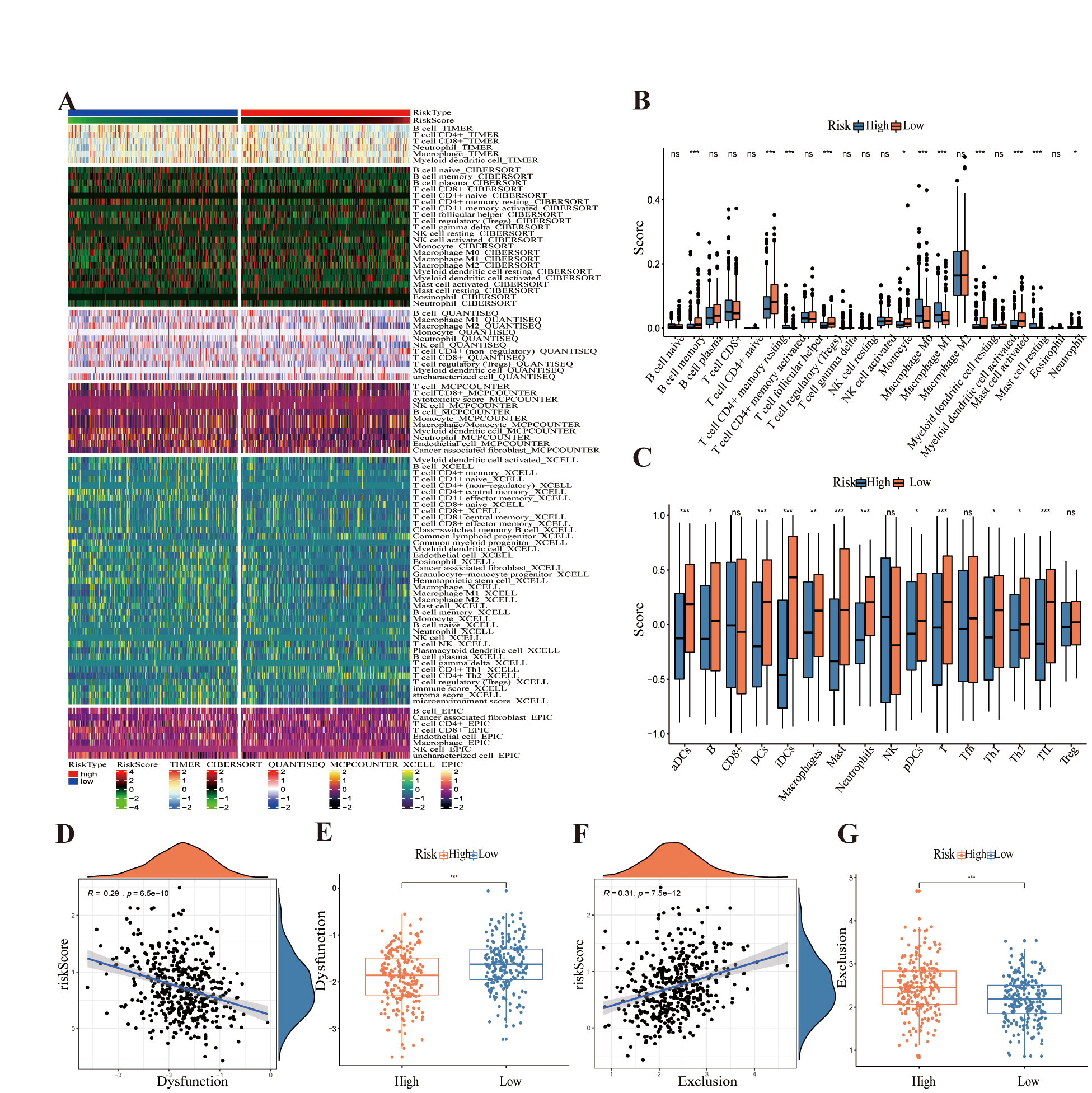


**Figure S11.** The characteristics in high and low CRG_score groups. (A) Six types of algorithms estimated immune cells infiltration in two CRG_score groups. (B) The estimated immune cells infiltration in two CRG_score groups by CIBERSORT. (C)The estimated immune cells infiltration in two CRG_score groups by ssGSEA.(D) The correlation between T cells dysfunction score and risk score. (E) T cells exclusion score in the two CRG_score subtypes. (F) The correlation between T cells exclusion score and risk score. (G) T cells exclusion score in the two CRG_score subtypes. *, **, and ***, represent P < 0.05, P < 0.01, and P < 0.001, respectively.


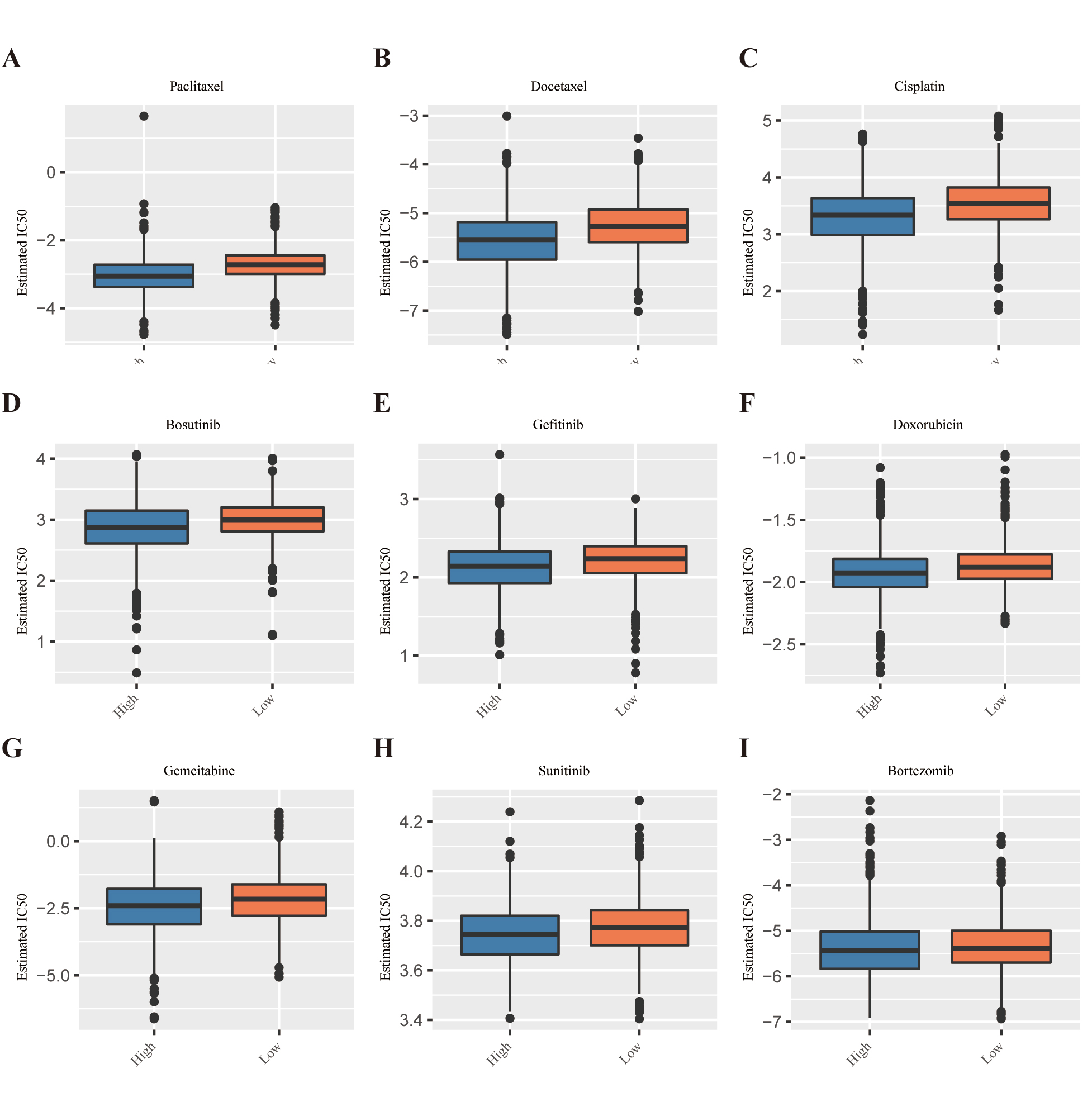


**Figure S12.** Drug sensitivity in different risk groups. The estimated IC50 between high and low CRG_score groups in (A)Paclitaxel; (B)Docetaxel; (C)Cisplatin; (D)Bosutinib; (E)Gefitinib; (F)Doxorubicin; (G)Gemcitabine; (H)Sunitinib; (I)Bortezomib.
